# Resonsive anion transport with a Hamilton receptor-based anionophore controlled by photo-activation and host–guest competitive inhibition†

**DOI:** 10.1039/d5sc02619a

**Published:** 2025-06-25

**Authors:** Manzoor Ahmad, Andrew Docker, Matthew J. Langton

**Affiliations:** a Chemistry Research Laboratory, Department of Chemistry, University of Oxford 12 Mansfield Road Oxford OX1 3TA UK matthew.langton@chem.ox.ac.uk; b Yusuf Hamied Department of Chemistry, University of Cambridge Lensfield Road Cambridge CB2 1EW, UK

## Abstract

Ion transport across biological membranes, facilitated by naturally occurring ion channels and pumps, is crucial for many biological functions. Many of these transport systems are gated, such that ion transport is regulated by a range of external stimuli, including light, small molecule ligand binding, and membrane potential. Synthetic ion transport systems, including those with similar gating mechanisms, have garnered significant attention due to their potential applications in targeted therapeutics as anticancer agents or to treat channelopathies. In this work, we report stimuli-responsive anion transporters based on dynamic hydrogen bonding interactions of hydroxyl-functionalised Hamilton-receptor-based anionophores. Caging of the hydroxyl groups with a light-responsive *ortho*-nitrobenzyl (ONB) moiety locks the amide protons through intramolecular hydrogen bonding, making them unavailable for anion binding and transport. Decaging with light reverses the hydrogen bonding pattern, rendering the amide protons available for anion binding and transport. Addition of a barbiturate ligand switches OFF the ion transport activity by blocking the anion binding cavity through competitive inhibition. OFF–ON–OFF reversible control over anion transport is therefore achieved using a combination of light and competitive small molecular ligand binding.

## Abstract 2

Stimuli-responsive anion transporters derived from hydroxyl-functionalised Hamilton-receptors are reported. Transport activity may be switched between on and off states, using photoactivation and competitive inhibition through ligand binding.

## Graphical abstract

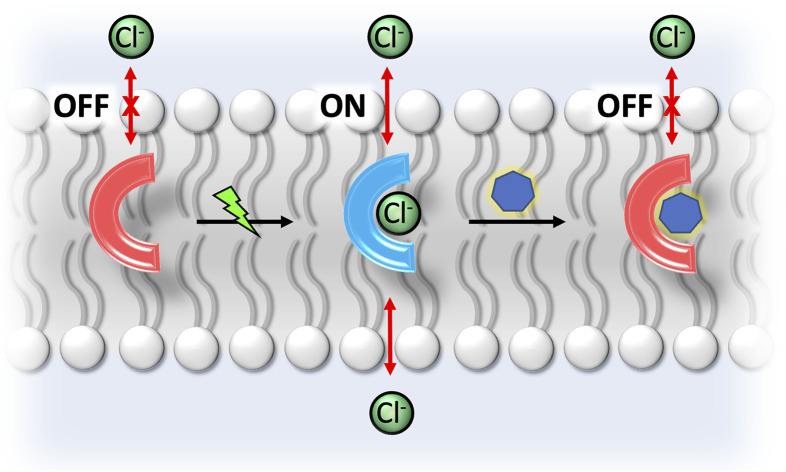

## Introduction

Biological systems are characterized by their precise regulation of chemical processes, energy production, and cellular homeostasis. A key component of these is the regulation of intracellular ion concentrations, which is achieved through naturally occurring ion channels and pumps, which in turn are tightly regulated by external stimuli such as small molecules, membrane potential and light.^1^ Indeed, the malfunction of ion transport proteins is associated with life-threatening diseases, such as cystic fibrosis, which arises from misregulated chloride transport.^2^ As such, synthetic supramolecular anionophores^3^ have attracted significant interest as novel treatments for these diseases, as well as for potential cancer chemotherapeutics.^4–8^ However, coupling anion transport activity in these systems with stimulus-responsive control for spatio-temporal targeted applications, as is the case for biological systems, remains rare.^9^ Multi-stimuli control offers the potential for tighter regulation of activity, but examples of this are rarer still.^10–13^ Whilst systems controlled by light,^14–23^ pH,^24–27^ enzymes,^13,28^ redox^29–32^ and membrane potential^33^ have been reported, achieving high levels of control to switch between fully inactive “OFF” states and ion-transporting “ON” states is particularly challenging.

Reversibly gated ionophores based on azobenzene and stilbene photoswitches may exhibit background transport activities because of incomplete photoconversion between the two isomers.^29^ Reversibly gated systems based on acylhydrazone and phenylhydrazone photoswitches have been developed by Talukdar and coworkers, and possess metastable states stabilized by intramolecular hydrogen bonding of the *Z* isomer.^11,12^ However, the use of an exogenous acid input for activation is highly undesirable for biological contexts.

Irreversibly-gated systems can provide very effective OFF–ON control over ion transport activity. Examples include the use of photo-responsive cages to block anion binding sites in anion carriers,^34,35^ control the self-assembly behaviour of channel-forming transporters,^36^ or control anionophore lipid bilayer membrane mobility.^37^ Other similar caged systems include the use of chalcogen bonding anionophores,^31,38^ Au(iii) caged systems,^30^ and enzyme-responsive carriers.^28^ Recently, we developed new approaches for controlling the transport activity utilizing caged anionophores.^13,39^ In these transporters, a caged OFF state features amide protons that are locked through intramolecular hydrogen bonding and unavailable for anion binding, while decaging using light, redox, or enzymes renders them available for anion binding and therefore transport. Although these systems were found to be effective in terms of their excellent OFF–ON activation behaviour, they lack a reversibly controlled response.

Despite the significant recent progress in the field of stimuli-controlled anionophore activity, one biological strategy that remains unexplored in the context of controllable synthetic ion carriers is that of competitive inhibition. Indeed, this highly effective mechanism allows for precise, reversible and controlled activity of ion transporters in nature. This approach has been exploited for the development of numerous therapeutics targeting ion channels, for example, those to treat heart arrhythmias by inhibiting cardiac sodium channels,^40^ and a handful of synthetic ion channels gated by ligand binding have been reported.^41–47^ We envisaged that competitive inhibition could conceivably be applied to anionophore design, wherein the addition of a correctly designed inhibitor forms a stable complex with the anion carrier, thereby blocking and turning ‘OFF’ anion transport activity.

**Fig. 1:**
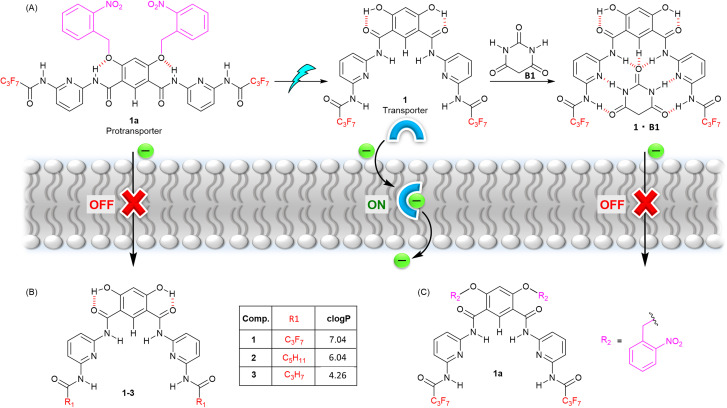
(A) Schematic representation of stimuli-responsive anion transport. (B) Chemical structure of transporters 1–3, with clogP values calculated using MarvinSketch. (C) Caged pro-transporter 1a.

Herein, we report a gated anion carrier transport system based on a Hamilton receptor^48^ incorporating a hydrogen bond switch mechanism, utilising both photo-activation and an unprecedented competitive inhibition mechanism to control its activity (Fig. 1). Caging of the hydroxy groups of the Hamilton receptor with *ortho*-nitrobenzyl (ONB) group inhibits anion transport by locking anion binding amide protons through intramolecular hydrogen-bonding. Decaging of ONB groups using light reverses the hydrogen bonding pattern, rendering amide N–H protons available for anion binding and transport. The transport activity can be subsequently turned OFF using a barbiturate ligand as an external stimulus, which competitively binds to the Hamilton receptor through multiple intermolecular hydrogen bonding interactions, thus inhibiting anion binding and transport.

## Results and discussion

### Design of the stimuli-responsive transporter platform

We identified a 4,6-dihydroxy-based Hamilton receptor as the key building block for the responsive anionophore platform (Fig. 1B). The Hamilton receptor provides the necessary 4,6-dihydroxy-isophthalamide core for anion binding and its transport across the lipid bilayer membrane, while preorganization *via* intramolecular hydrogen bonding between the hydroxyl hydrogen bond donors and the adjacent carbonyl groups was expected to enhance anion binding and transport. Alkylation of these hydroxy groups in a 4,6-dihydroxy-isophthalamide anion receptor is known to disrupt this pattern, leading to an *anti*–*anti* arrangement of the amides and a decrease in anion binding affinity.^49^ Ren and coworkers, and our group, have previously developed stimuli-responsive transport systems using a similar approach with alkyl-functionalized dihydroxy isophthalamide derivatives, in which photo-dealkylation of the hydroxyl groups generates the hydroxyl form, facilitating H^+^/Cl^−^ or NO_3_^−^/Cl^−^ transport across lipid bilayer membranes.^39,50,51^

In our current work, we have targeted a Hamilton receptor derived from a 4,6-dihydroxy-isophthalamide scaffold as the key anionophore motif, which we anticipated would provide access to the corresponding *ortho*-nitrobenzyl (ONB) stimuli-responsive pro-anionophore upon alkylation of the hydroxy groups with ONB moieties (Fig. 1C). Subsequent photo-decaging would generate the active transporter, which could be deactivated again by competitive binding of a barbiturate ligand added as an external stimulus (Fig. 1A). Barbiturate ligands are known to bind to Hamilton receptors strongly though multiple intermolecular hydrogen bonding interactions.^52,53^ Furthermore, varying the amide substitution of the Hamilton receptor's pyridine ring was expected to fine-tune the lipophilicities of the active transporters, in turn modulating their membrane permeabilities and therefore transport activities (Fig. 1B).

### Synthesis and anion binding experiments

**Fig. 2:**
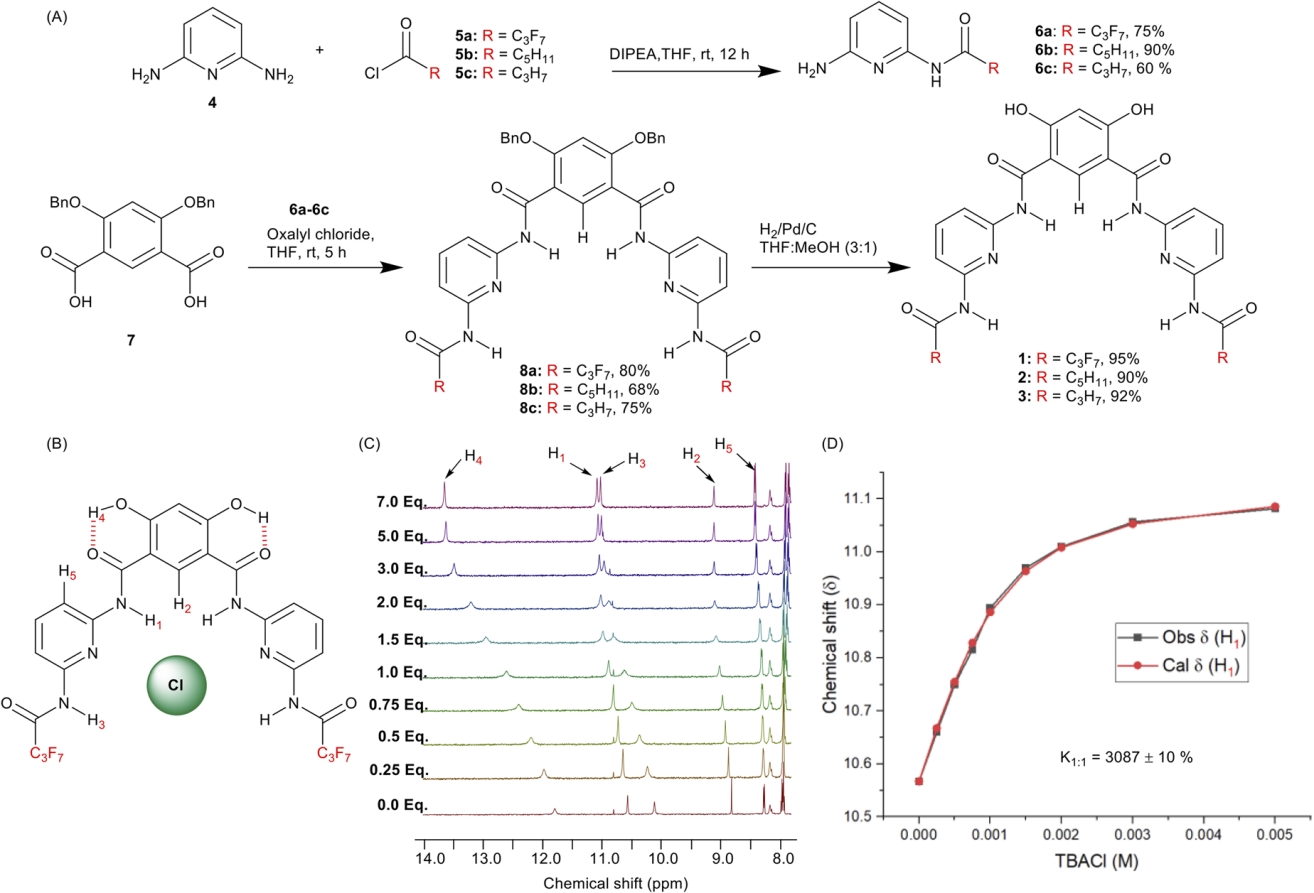
(A) Synthesis of transporters 1–3. (B) Chemical structure of transporter 1 complexed to chloride. (C) ^1^H NMR titration spectra for 1 (2.5 mM in THF-*d*_8_) with stepwise addition of TBACl in THF-*d*_8_. Protons assigned in panel B, and the equivalents of added TBACl are shown on the stacked spectra. (D) The plot of chemical shift (*δ*) of proton H_1_*vs.* concentration of TBACl added for transporter 1, fitted to a 1 : 1 binding model using BindFit.

To achieve the synthesis of the transporters 1–3, 2,5-diaminopyridine 4 was converted into mono-amide derivatives 6a–6c upon reacting with acyl chlorides 5a–5c. Compounds 6a–6c were coupled with di-benzylated isophthalic acid 7,^50^ to furnish debenzylated Hamilton receptor derivatives 8a–8c. Finally, compounds 8a–8c were debenzylated upon reaction with hydrogen in the presence of palladium on carbon to furnish the anionophores 1–3 in excellent yields (Fig. 2A).

To investigate the anion binding capabilities of compound 1, ^1^H NMR titration studies were performed in THF–*d*_8_. Addition of increasing equivalents of either tetrabutylammonium chloride (TBACl) or tetrabutylammonium nitrate (TBANO_3_) to receptor 1 lead to downfield chemical shift of the amide N–H_1_, N–H_3_ and C_Ar_–H_2_ protons, respectively (Fig. 2C, S23 and S25†), indicative of N–H_1_⋯A^−^, N–H_3_⋯A^−^, and C_Ar_–H_2_⋯A^−^ hydrogen bonding interactions and providing evidence for the pre-organised binding conformation (Fig. 2B). Analysis of the generated binding isotherms using Bindfit^54^ determined the 1 : 1 host : guest association constant (*K*_a(1 : 1)_/Cl^−^) of 3087 M^−1^ ± 10% and (*K*_a(1 : 1)_/NO_3_^−^) of 2639 M^−1^ ± 8%, respectively (Fig. 2D, S24 and S26†).

Anion binding studies could not be performed for receptors 2 and 3 because of their insolubility in THF–*d*_8_ and other polar organic solvents. In addition to the chloride-induced chemical shift perturbations of H_1_, H_2_ and H_3_, further evidence for the pre-organised conformation was obtained from ^1^H–^1^H ROESY NMR spectroscopy in which through-space correlations were observed between protons H_1_ and H_2_ (Fig. S21†). This is consistent with previous studies highlighting the role of the intra-molecular hydrogen bonds in pre-organising such 4,6-dihydroxy-isophthalamide scaffolds, including crystallographic structural data of related systems.^39,49^

### Transmembrane ion transport experiments

**Fig. 3:**
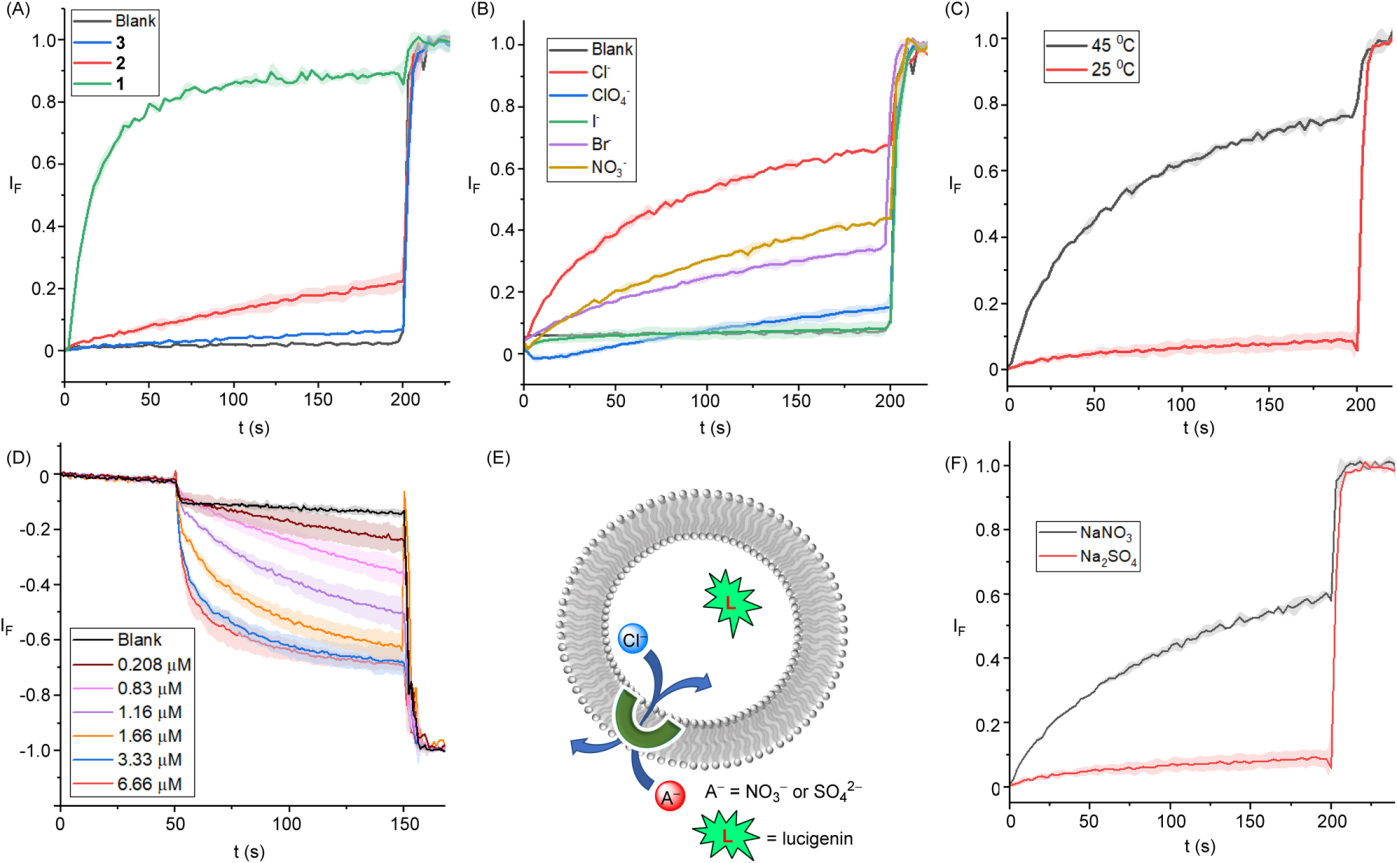
(A) Ion transport activity comparison of 1–3 (1.34 mol%) across POPC-LUVs⊃HPTS. (B) Dependence of activity of 1 (0.193 mol%) on external anion NaX (where X^–^ = Cl^−^, Br^−^, I^−^, NO_3_^−^, and ClO_4_^−^). (C). Ion transport activity of 1 (0.33 mol%) across DPPC-based vesicles at 25 °C and 45 °C temperatures, respectively. (D) Ion transport activity of 1 across POPC-LUVs⊃lucigenin. (E) Schematic representation of chloride efflux with either extravesicular SO_4_^2−^ and NO_3_^−^ ions in the lucigenin assay. (F) Ion transport activity of 1 (3.0 mol%) in the presence of external SO_4_^2−^ and NO_3_^−^ ions.

The ion transport activities of the compounds 1–3 were evaluated using large unilamellar vesicles (LUVs) fluorescence assays. 1-Palmitoyl-2-oleoyl-sn-glycero-3-phosphocholine vesicles (POPC LUVs) containing the pH-sensitive fluorophore 8-hydroxy pyrene-1,3,6-trisulfonic acid trisodium salt (HPTS, 1 mM) were prepared, containing 100 mM of NaCl and 10 mM HEPES buffer at pH 7. Subsequently, a pH gradient (pH_in_ = 7.0 and pH_out_ = 7.8) was created across the LUV membrane by the addition of a pulse of NaOH (5 mM) in the extravesicular solution. After addition of compounds 1–3, the dissipation of the pH gradient through OH^−^/Cl^−^ antiport (or the functionally equivalent H^+^/Cl^−^ symport) was monitored by recording the change in fluorescence intensity at *I*_rel_ (*λ*_em_ = 510 nm), with time following excitation at *λ*_ex_ = 405/465. At the end of each experiment, excess detergent (Triton X-100) was added to lyse the vesicles for calibration of the emission intensity. Significant ion transport was observed for all transporters with the activity sequence of 1 > 2 > 3. The activity of the three transporters at 1.34 mol% loading (with respect to lipid) is shown in Fig. 3A, and the corresponding dose-response curves are given in the ESI (Fig. S29–S34†). Hill analysis furnished the *EC*_50_ values of 0.032 μM ± 0.002 (0.096 ± 0.005 mol%) for 1, 7.1 μM ± 0.4 (22.9 ± 0.1 mol%) for 2, and 23.6 μM ± 1.9 (76.1 ± 6.0 mol%) for 3, respectively. Hill coefficients (*n*) were found to be ∼1 for all cases, suggesting the involvement of a 1 : 1 receptor–anion complex in the transmembrane anion transport process. The above activity sequences of 1 > 2 > 3 is most likely determined by the varying lipophilicity of the transporters, as the substituents attached to fine-tune their lipophilicities are not anticipated to significantly affect the anion binding properties of these transporters. The compound 1 with the optimum clogP value furnishes the best transport activity.

Chloride transport by the best-performing transporter 1 was subsequently studied using lucigenin-based LUV assays. LUVs entrapping lucigenin – a fluorophore quenched by chloride – were prepared in 200 mM NaNO_3_ buffered to pH 6.5 with 10 mM phosphate. Transporter 1 was then added in a 20 μL aliquot of DMF at varying concentrations before a Cl^−^/NO_3_^−^gradient was created by adding NaCl (33.3 mM) in the extravesicular buffer. The influx of chloride was evaluated by monitoring the rate of change in the fluorescence intensity (*λ*_ex_ = 455 nm and *λ*_em_ = 535 nm), and at the end of each experiment, excess detergent (Triton X-100) was added to lyse the vesicles for calibration of the emission intensity. Significant quenching was observed for the lucigenin after the addition of transporter 1. The dose-dependent Cl^−^ influx by 1 is shown in Fig. 3D. Hill analysis furnished *EC*_50_ value of 0.635 ± 0.94 μM with a Hill coefficient value of ∼1, consistent with that obtained in the above-mentioned HPTS pH dissipation experiments (Fig. S37†).

Mechanistically, the disruption of the pH gradient across the above POPC-LUVs⊃HPTS can occur through: (a) H^+^/X^−^ symport, (b) OH^−^/X^−^ antiport, (c) H^+^/M^+^ antiport, or (d) OH^−^/M^+^ symport transport modes. However, using intracellular NaCl and an iso-osmolar extravesicular M^+^/Cl^−^ salt (where M^+^ = Li^+^, Na^+^, K^+^, Rb^+^, and Cs^+^), no change in ion transport activity was observed, which rules out the possibility of H^+^/M^+^ antiport and OH^−^/M^+^ symport modes (Fig. S35†). Furthermore, changing the extravesicular Na^+^/X^–^ salt (X^–^ = Cl^−^, Br^−^, I^−^, NO_3_^−^ and ClO_4_^−^) resulted in significant modulation of the ion transport activity (Fig. 3B), demonstrating the role of anions in the overall transport process, and hence suggest that the ion transport process can occur through either OH^−^/Cl^−^ antiport or H^+^/Cl^−^ symport modes. Similarly, inactivity of the system when the chloride anions in the buffer were exchanged for gluconate – a large hydrophilic anion too hydrophilic to be transported – further confirmed that the transport in the presence of chloride is cation independent, occurring *via* Cl^−^/OH^−^ antiport (or Cl^−^/H^+^ symport) and not *via* a cation dependent H^+^/Na^+^ antiport mechanism (Fig. S36†).

Finally, an antiport mode of transport was validated for Cl^−^/NO_3_^−^ exchange in the lucigenin-based fluorescence assays by exchanging the nitrate anion for the more hydrophilic dianionic sulfate. Lucigenin was encapsulated within the liposomes containing NaCl (200 mM) buffered at a pH of 6.5 with 10 mM phosphate. The ion transport activity was monitored in these LUVs suspended in either buffered NaNO_3_ (200 mM) or Na_2_SO_4_ (200 mM). The transport activity was determined by recording the change in the lucigenin emission, *I*_f_ (*λ*_em_ = 535 nm), with time following excitation at *λ*_ex_ = 460 nm. Significant transport activity was observed in the presence of NaNO_3_, which was absent in the presence of Na_2_SO_4_ (Fig. 3F). The above results suggest the Cl^−^/NO_3_^−^ antiport mode of ion transport dominates in this assay, rather than H^+^/Cl^−^ symport, because in the latter no change in transport rates would be expected upon changing the anion, whilst for antiport, sulfate is highly hydrophilic and poorly transported compared to nitrate. Conducting analogous experiments in dipalmitoylphosphatidylcholine (DPPC) LUVs provided evidence for a mobile carrier mechanism. Inactivity at 25 °C, and restoration of activity at 45 °C, which is above the gel–liquid phase transition temperature for DPPC (*T*_m_ = 41 °C), is indicative of a mobile carrier process, rather than transport mediated by self-assembly into an ion channel, the activity of which would be typically expected to be independent of the lipid phase (Fig. 3C).

### Synthesis and anion binding experiments of caged pro-transporter 1a

**Fig. 4:**
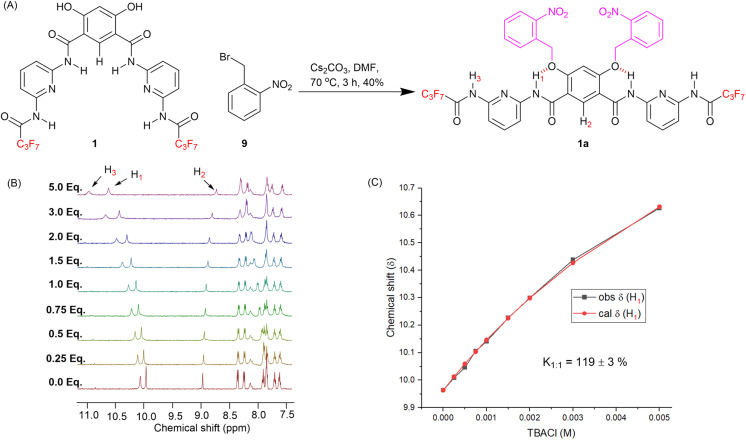
(A) Synthesis of pro-transporter 1a. (B) ^1^H NMR titration spectra for 1a (2.5 mM in THF-*d*_8_) with stepwise addition of TBACl in THF-*d*_8_. The equivalents of added TBACl are shown on the stacked spectra. (C) Plot of chemical shift (*δ*) of H_1_ proton *vs.* concentration of TBACl added for transporter 1, and fit to a 1 : 1 binding model.

Compound 1, with optimum transport capabilities, was selected for caging with ONB photocleavable groups. Alkylation of 1 with 1-(bromomethyl)-2-nitrobenzene gave the corresponding light-responsive pro-transporter 1a (Fig. 4A). Previous studies have demonstrated that such alkylation of the hydroxyl groups of the 4,6-dihydroxy-isophthalamide motif reverses the intra-molecular hydrogen bonding pattern, to adopt a conformation in which the amide NH protons interact with the ether O atoms, and are not available for anion binding.^13,39,49^ Data suggesting 1a adopts a similar conformation (Fig. 4A) was obtained from ROESY NMR and chloride binding titration experiments. ^1^H–^1^H ROESY interactions were observed between amide protons H_1_ and protons on the *ortho*-nitrobenzyl cages (Fig. S22†), as well as with H_2_, confirming the proposed conformation with intra-molecular hydrogen bonding interactions is present in solution, and that the system is in dynamic equilibrium. A ^1^H NMR chloride binding titration experiment with 1a in THF-*d*_8_ revealed minimal changes in the chemical shift of the amide protons upon addition of TBACl (Fig. 4B and S27†), which demonstrates the lack of chloride binding and suggests 1a predominantly adopts the conformation with intra-molecular amide NH–O hydrogen bonds, which renders them less available for anion recognition. Using Bindfit, a 1 : 1 host–guest association constant (*K*_a(1 : 1)_/Cl^−^) value of 120 M^−1^ ± 3% was determined (Fig. 4C and S28†), which is dramatically reduced compared to the association constant value of 3087 M^−1^ ± 10% for the preorganised, decaged transporter 1.

### Stimuli-responsive ion transport experiments

Before proceeding to stimulus-responsive ion transport studies, photoactivation of pro-transporter 1a and binding studies of transporter 1 with barbiturate ligands were performed in the solution phase. For 1a, a 1 mM solution of the pro-transporter in DMSO-*d*_6_ was subjected to photoirradiation using a 405 nm LED and analysed by ^1^H NMR experiments. Complete decaging of 1a to generate 1 was observed after 50 minutes of irradiation under these conditions (Fig. S40†). To monitor the binding of barbiturate ligand to Hamilton receptor 1, UV-vis absorption studies were performed. Aliquots of barbiturate solution of B1 (200 mM in DMF) were added to the receptor solution of 1 (6.6 μM in DMF), and absorption spectra were recorded. Addition of barbiturate ligand B1 to receptor 1 lead to a reduction in absorbance of the band at 363 nm corresponding to the free receptor, and an increase in absorbance at 320 nm corresponding to the host–guest complex (Fig. S42†). These changes confirmed the binding of the barbiturate ligand with the transporter molecule 1. The data could be fitted to a 1 : 1 host–guest binding model, affording an association constant value of (*K*_(1 : 1)_/B1) = 141 M^−1^ ± 5% (Fig. S43†). Titration experiments with TBACl revealed that the analogous Cl^−^ association constant in DMF is approximately one order of magnitude lower (*K*_a(1 : 1)_/Cl^−^ = 9 M^−1^ ± 14%, Fig. S44 and S45†).

**Fig. 5:**
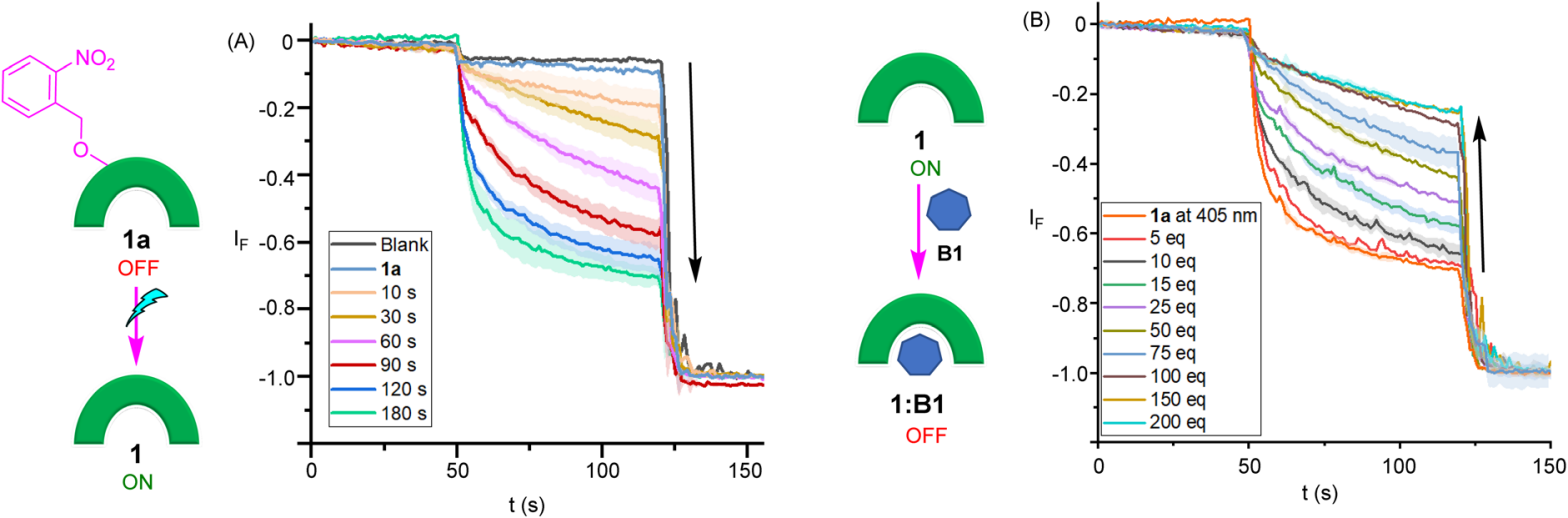
(A) Ion transport activities across POPC-LUVs⊃lucigenin after photo-irradiating 1a at 405 nm light using an LED (1 W) and (B) treating 1a with light for 5 minutes followed by the addition of barbiturate ligand B1.

With evidence of efficient activation of pro-transporter 1a to provide 1, as well as barbiturate ligand binding to transporter 1 in the solution phase in hand, stimulus-responsive OFF–ON transport was evaluated using lucigenin-containing LUVs in phosphate buffered NaNO_3_ solution. Pro-transporter 1a was added in an aliquot of DMF (final concentration of 3.0 mol% with respect to lipid), followed by adding NaCl (33.3 mM) in the extravesicular buffer to generate a Cl^−^/NO_3_^−^gradient. No significant changes were observed in the lucigenin fluorescence, indicating that the pro-transporter 1a is inactive at this concentration, consistent with results from ^1^H NMR chloride binding experiments, thus achieving an effective OFF state. Pro-transporter 1a in phosphate buffered NaNO_3_ solution in a cuvette was then subjected to irradiation with a 1 W 405 nm LED, before adding LUVs and initiating ion transport by the addition of a chloride pulse. Photo-activation of 1a (3.0 mol%) resulted in efficient activation, achieving comparable activity to a sample of 1 after 180 s of irradiation, indicative of near quantitative decaging under these conditions (Fig. 5A). For deactivation, 1a (3.0 mol%) was initially photo-irradiated in the phosphate buffered NaNO_3_ solution with the 405 nm LED for 5 minutes to generate 1, and subsequently barbiturate B1 in DMF was added in with increasing equivalents and ion transport activity was monitored. Addition of barbiturate ligand B1 (5–200 eq.) led to the significant deactivation of the ion transport (Fig. 5B). This deactivation was attributed to host–guest complex formation between the transporter 1 and the barbiturate ligand which results in the blockage of the anion binding cavity and stops the ion transport activity. To the best of our knowledge, this represents the first example of competitive inhibition of an ionophore by ligand binding.

## Conclusions

In conclusion, we have developed stimuli-responsive anion transporters based on hydrogen bonding interactions of hydroxyl-functionalised Hamilton receptor-based anionophores. Caging of the hydroxyl groups with light-responsive *ortho*-nitrobenzyl (ONB) causes the receptor to adopt a conformation in which the amide NH protons form intra-molecular interactions with the benzyl-ether O atoms, rendering them unavailable for anion binding and transport. Decaging with light reverses the hydrogen bonding pattern, such that the amide protons are available for anion binding and anion transport. The ion transport activity could be turned off again upon competitive binding of a barbiturate ligand to the Hamilton-receptor binding cavity. The combination of light and small molecule ligand binding thus provides a dual stimuli-responsive system enabling OFF–ON–OFF control over transport using an unprecedented competitive inhibition mechanism to regulate the anionophore activity.

## Author contributions

M. A. performed the synthesis and characterization of the compounds, as well as the anion binding and transport studies and stimuli-responsive investigations. A. D. performed the synthesis and characterization of some of the compounds. M. A. wrote the first draft of the manuscript, which was edited by all authors. M. J. L. supervised the project.

## Conflicts of interest

There are no conflicts to declare.

## Supplementary Material

SC-OLF-D5SC02619A-s001

## Data Availability

All data supporting the findings of this study are available within the article and ESI.†

## References

[cit1] Alberts B. , Johnson A., Lewis J., Raff M., Roberts K. and Walter P., Molecular Biology of the Cell, 4th edn, 2002

[cit2] Choi J. Y., Muallem D., Kiselyov K., Lee M. G., Thomas P. J., Muallem S. (2001). Nature.

[cit3] Davis J. T., Gale P. A., Quesada R. (2020). Chem. Soc. Rev..

[cit4] Busschaert N., Wenzel M., Light M. E., Iglesias-Hernández P., Pérez-Tomás R., Gale P. A. (2011). J. Am. Chem. Soc..

[cit5] Moore S. J., Haynes C. J. E., González J., Sutton J. L., Brooks S. J., Light M. E., Herniman J., Langley G. J., Soto-Cerrato V., Pérez-Tomás R., Marques I., Costa P. J., Félix V., Gale P. A. (2013). Chem. Sci..

[cit6] Ko S.-K., Kim S. K., Share A., Lynch V. M., Park J., Namkung W., Van Rossom W., Busschaert N., Gale P. A., Sessler J. L., Shin I. (2014). Nat. Chem..

[cit7] Saha T., Hossain M. S., Saha D., Lahiri M., Talukdar P. (2016). J. Am. Chem. Soc..

[cit8] Busschaert N., Park S.-H., Baek K.-H., Choi Y. P., Park J., Howe E. N. W., Hiscock J. R., Karagiannidis L. E., Marques I., Félix V., Namkung W., Sessler J. L., Gale P. A., Shin I. (2017). Nat. Chem..

[cit9] Langton M. J. (2021). Nat. Rev. Chem..

[cit10] Peters A. D., Borsley S., Della Sala F., Cairns-Gibson D. F., Leonidou M., Clayden J., Whitehead G. F. S., Vitórica-Yrezábal I. J., Takano E., Burthem J., Cockroft S. L., Webb S. J. (2020). Chem. Sci..

[cit11] Ahmad M., Chattopadhayay S., Mondal D., Vijayakanth T., Talukdar P. (2021). Org. Lett..

[cit12] Ahmad M., Mondal D., Roy N. J., Vijayakanth T., Talukdar P. (2022). ChemPhotoChem.

[cit13] Ahmad M., Johnson T. G., Flerin M., Duarte F., Langton M. J. (2024). Angew. Chem., Int. Ed..

[cit14] Choi Y. R., Kim G. C., Jeon H.-G., Park J., Namkung W., Jeong K.-S. (2014). Chem. Commun..

[cit15] Ahmed M., Metya S., Das A., Talukdar P. (2020). Chem. Eur. J..

[cit16] Ahmad M., Sarkar S., Bhogade R., Mondal A., Mondal D., Mondal J., Talukdar P. (2025). Nanoscale.

[cit17] Johnson T. G., Sadeghi-Kelishadi A., Langton M. J. (2022). J. Am. Chem.
Soc..

[cit18] Kerckhoffs A., Langton M. J. (2020). Chem. Sci..

[cit19] Wang W.-Z., Huang L.-B., Zheng S.-P., Moulin E., Gavat O., Barboiu M., Giuseppone N. (2021). J. Am. Chem. Soc..

[cit20] Kerckhoffs A., Ahmad M., Langton M. J. (2024). Chem.–Eur. J..

[cit21] Wezenberg S. J., Chen L.-J., Bos J. E., Feringa B. L., Howe E. N. W., Wu X., Siegler M. A., Gale P. A. (2022). J. Am. Chem. Soc..

[cit22] Bos J. E., Siegler M. A., Wezenberg S. J. (2024). J. Am. Chem. Soc..

[cit23] Grählert E., Langton M. J. (2024). Angew. Chem., Int. Ed..

[cit24] Howe E. N. W., Busschaert N., Wu X., Berry S. N., Ho J., Light M. E., Czech D. D., Klein H. A., Kitchen J. A., Gale P. A. (2016). J. Am. Chem. Soc..

[cit25] Busschaert N., Elmes R. B. P., Czech D. D., Wu X., Kirby I. L., Peck E. M., Hendzel K. D., Shaw S. K., Chan B., Smith B. D., Jolliffe K. A., Gale P. A. (2014). Chem. Sci..

[cit26] Roy A., Biswas O., Talukdar P. (2017). Chem. Commun..

[cit27] Shinde S. V., Talukdar P. (2017). Angew. Chem., Int. Ed..

[cit28] Choi Y. R., Lee B., Park J., Namkung W., Jeong K.-S. (2016). J. Am. Chem. Soc..

[cit29] Ahmad M., Gartland S. A., Langton M. J. (2023). Angew. Chem., Int. Ed..

[cit30] Fares M., Wu X., Ramesh D., Lewis W., Keller P. A., Howe E. N. W., Pérez-Tomás R., Gale P. A. (2020). Angew. Chem., Int. Ed..

[cit31] Zhou B., Gabbaï F. P. (2020). Chem. Sci..

[cit32] Docker A., Johnson T. G., Kuhn H., Zhang Z., Langton M. J. (2023). J. Am. Chem. Soc..

[cit33] Wu X., Small J. R., Cataldo A., Withecombe A. M., Turner P., Gale P. A. (2019). Angew. Chem., Int. Ed..

[cit34] Salunke S. B., Malla J. A., Talukdar P. (2019). Angew. Chem., Int. Ed..

[cit35] Ahmad M., Roy N. J., Singh A., Mondal D., Mondal A., Vijayakanth T., Lahiri M., Talukdar P. (2023). Chem. Sci..

[cit36] Bao C., Ma M., Meng F., Lin Q., Zhu L. (2015). New J. Chem..

[cit37] Bickerton L. E., Langton M. J. (2022). Chem. Sci..

[cit38] Park G., Gabbaï F. P. (2020). Chem. Sci..

[cit39] Ahmad M., Flerin M., Tay H. M., Thompson A. L., Duarte F., Langton M. J. (2024). Nanoscale.

[cit40] Ramos E., O'Leary M. E. (2004). J. Physiol..

[cit41] Gorteau V., Perret F., Bollot G., Mareda J., Lazar A. N., Coleman A. W., Tran D. H., Sakai N., Matile S. (2004). J. Am. Chem. Soc..

[cit42] Talukdar P., Bollot G., Mareda J., Sakai N., Matile S. (2005). Chem. Eur. J..

[cit43] Tedesco M. M., Ghebremariam B., Sakai N., Matile S. (1999). Angew. Chem., Int. Ed..

[cit44] Muraoka T., Shima T., Hamada T., Morita M., Takagi M., Tabata K. V., Noji H., Kinbara K. (2012). J. Am. Chem. Soc..

[cit45] Wilson C. P., Webb S. J. (2008). Chem. Commun..

[cit46] Boccalon M., Iengo E., Tecilla P. (2012). J. Am. Chem. Soc..

[cit47] Haynes C. J. E., Zhu J., Chimerel C., Hernández-Ainsa S., Riddell I. A., Ronson T. K., Keyser U. F., Nitschke J. R. (2017). Angew. Chem., Int. Ed..

[cit48] Chang S. K., Hamilton A. D. (1988). J. Am. Chem. Soc..

[cit49] Santacroce P. V., Davis J. T., Light M. E., Gale P. A., Iglesias-Sánchez J. C., Prados P., Quesada R. (2007). J. Am. Chem. Soc..

[cit50] Zhong Q., Cao Y., Xie X., Wu Y., Chen Z., Zhang Q., Jia C., Wu Z., Xin P., Yan X., Zeng Z., Ren C. (2024). Angew. Chem., Int. Ed..

[cit51] Ahmad M., Muir A., Langton M. J. (2024). ACS Omega.

[cit52] Tron A., Rocher M., Thornton P. J., Tucker J. H. R., McClenaghan N. D. (2015). Asian J. Org. Chem..

[cit53] Adhikari S., Datta A., Saha I., Ghosh K. (2024). Coord. Chem. Rev..

[cit54] http://app.supramolecular.org/bindfit/

