# Supplementary material for: Resonsive anion transport with a Hamilton receptor-based anionophore controlled by photo-activation and host–guest competitive inhibition
Source: Chem Sci. 2025 Jun 25;16(30):13715–22. doi: 10.1039/d5sc02619a (PMC12211230; doi:10.1039/d5sc02619a)
Supplement: SC-016-D5SC02619A-s001 [file SC-016-D5SC02619A-s001.pdf]

Supplementary Information (SI) for Chemical Science.
This journal is © The Royal Society of Chemistry 2025

Supporting Information for

Responsive anion transport with a Hamilton receptor-based anionophore controlled by photo-activation and host-guest competitive inhibition

Manzoor Ahmad,[a] Andrew Docker,[a,b] and Matthew J. Langton,[a*]

[a]*Chemistry Research Laboratory, Department of Chemistry, University of Oxford 12 Mansfield Road, Oxford, OX1 3TA, UK. E-mail:* *matthew.langton@chem.ox.ac.uk*

[b] *Yusuf Hamied Department of Chemistry, University of Cambridge, Lensfield Rd, Cambridge CB2 1EW*

Contents

Contents 1
I. Materials and methods 2
II. Synthesis 3
III. NMR Spectra 8
IV. Anion Binding Studies 20
V. Ion transport studies 23
VI. Solution phase photocleavage and ligand binding studies 31
VII. Stimulus-responsive ion transport studies 36
VIII. References 36

I. Materials and methods

All reagents and solvents were purchased from commercial sources and used without further purification. Lipids were purchased from Avanti Polar Lipids and used without further purification. Column chromatography was carried out on Merck® silica gel 60 under a positive pressure of nitrogen. Where mixtures of solvents were used, ratios are reported by volume. NMR spectra were recorded on a Bruker AVIII 400, Bruker AVII 500 (with cryoprobe) and Bruker AVIII 500 spectrometers. Chemical shifts are reported as δ values in ppm. Mass spectra were carried out on a Waters Micromass LCT and Bruker microTOF spectrometers. UV-Vis spectra were recorded on a V-770 UV-Visible/NIR Spectrophotometer equipped with a Peltier temperature controller and stirrer, using quartz cuvettes of 1 cm path length. Fluorescence spectroscopic data were recorded using Agilent fluorescence spectrophotometer, equipped with Peltier temperature controller and stirrer. Experiments were conducted at 25°C unless otherwise stated. Vesicles were prepared as described below using Avestin "LiposoFast" extruder apparatus, equipped with polycarbonate membranes with 200 nm pores. GPC purification of vesicles was carried out using GE Healthcare PD-10 desalting columns prepacked with Sephadex G 25 medium. HPLC analysis were carried out using a Thermo Scientific Vanquish Core HPLC and a C-18 reverse phase column (Ascentis, 5 µm, 15 cm x 4.6 mm).

Abbreviations

DCM: Dichloromethane; **DMF:** *N*, *N*-Dimethylformamide; **THF:** Tetrahydrofuran; **DMSO:** Dimethylsulfoxide; **DPPC:** 1,2-dipalmitoyl-sn-glycero-3-phosphocholine; **EYPG:** egg-yolk phosphatidylglycerol; **HRMS:** High resolution mass spectrometry; **LUVs:** large unilamellar vesicles; **MeOH:** Methanol; **POPC:** 1-palmitoyl-2-oleoyl-sn-glycero-3-phosphocholine; rt: Room temperature; **TBACl:** Tetrabutylammonium chloride.

II. Synthesis

Synthesis of mono-amide derivatives 6a-6c:

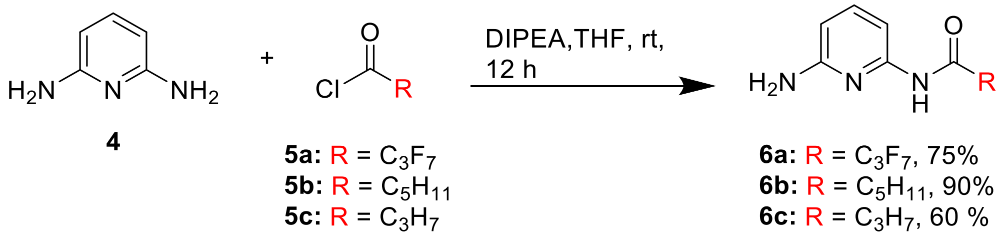

Scheme 1. Chemical synthesis of compounds **6a-6c**.

Compound **6b**[1] and **6c**[2] were prepared according to reported literature procedures.

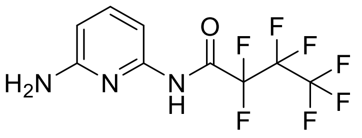

6a

$C_9H_6F_7N_3O$

***N*-(6-aminopyridin-2-yl)-2,2,3,3,4,4,4-heptafluorobutanamide (6a):** In a 100 mL round bottom flask, 2,6-diaminopyridine **4** (1.00 g, 9.10 mmol, 1 eq) and (DIPEA 1.18 g, 9.1 mmol, 1 eq) were dissolved in dry THF (20 mL). A solution of **5a** (2.13 g, 9.1 mmol, 1 eq) in dry THF (5 mL) was added dropwise over a period of 1 h. After the addition was completed the reaction mixture was stirred for 12 h at room temperature under nitrogen atmosphere. After the completion of the reaction, the solvent was evaporated through a rotary evaporator, and the crude mixture was extracted with ethyl acetate (3 × 50 mL). The organic layers were dried with anhydrous magnesium sulfate, filtered, and concentrated under reduced pressure to give the crude product. The crude mixture was then purified by column chromatography over silica gel (*Eluent:* 30% ethyl acetate in hexane) to furnish **6c** as a white solid. Yield: 75%, 2.10 g. **^{1}H NMR (400 MHz, CDCl$_3$)** δ 8.25 (s, 1H), 7.50 – 7.37 (m, 2H), 6.30 (dd, J = 7.3, 1.5 Hz, 1H), 4.37 (s, 2H); **^{13}C NMR (151 MHz, CDCl$_3$)** δ 157.41, 155.2 (t, J = 26.4 Hz), 147.42, 140.59, 117.5 (tq, J = 288 Hz, J = 33 Hz), 110.5-106.2 (m), 106.60, 103.89. **^{19}F NMR (565 MHz, CDCl$_3$)** δ -80.52 (t, J = 8.5 Hz), -120.52 (q, J = 8.5 Hz), -126.69 (app. s); **HRMS (ESI) *m/z*:** Calcd. for $C_9H_7F_7N_3O$ $[M+H]^+$, 306.0472; Found 306.0476.

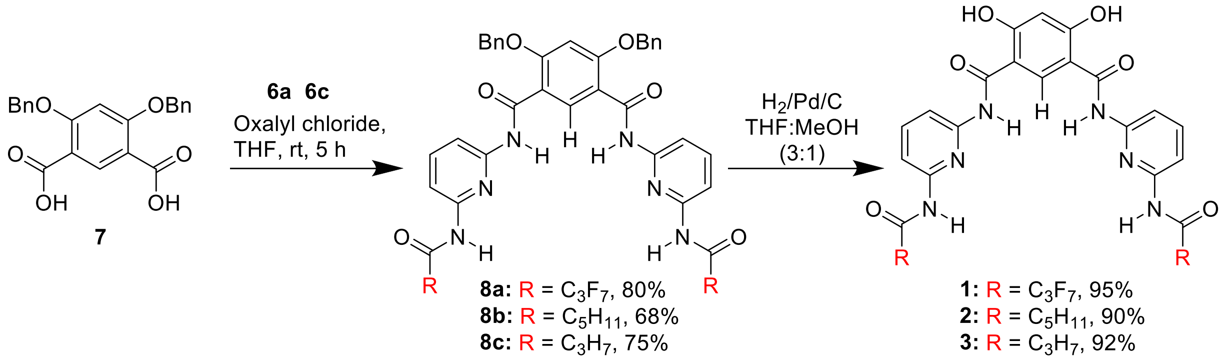

Scheme 2. Chemical synthesis of compounds **1-3**.

General procedure A: synthesis of compounds 8a-8c:

In an oven dry 25 mL round bottom flask, 4,6-bis(benzyloxy)isophthalic acid **7** (100 mg, 0.26 mmol, 1 eq) was dissolved in dry tetrahydrofuran (20 mL). Three drops of dry DMF were added to the mixture,

before oxalyl chloride (300 mg, 2.64 mmol, 10.0 eq) was added and reaction was stirred at rt for 5 h. After the completion of the reaction, solvent and excess of oxalyl chloride were removed using rotary evaporator. The crude was dissolved in dry tetrahydrofuran (10 mL). **6a-6c** (2 eq) and DIPEA (68.0 mg, 0.52 mmol, 2 eq) was added and the reaction mixture was allowed to stir at rt for 3 h. After the completion of the reaction, excess of solvent was removed under rotary evaporator and the crude was extracted with ethyl acetate (50 mL × 3). The organic layers were collected and dried over magnesium sulfate. The solvent was removed and the crude mixture was purified by column chromatography over silica gel to furnish **8a-8c** 68-80% yields.

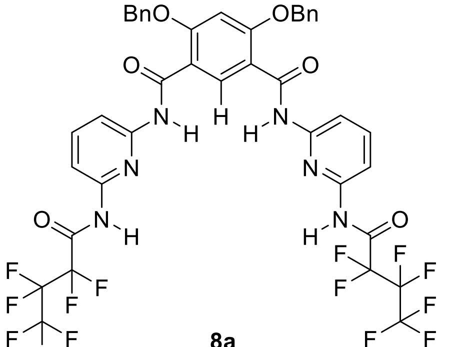

8a

$C_{40}H_{26}F_{14}N_6O_6$

4,6-bis(benzyloxy)-N1,N3-bis(6-(2,2,3,3,4,4,4-heptafluorobutanamido)pyridin-2-yl)isophthalamide (8a): This compound was synthesized by reacting 4,6-bis(benzyloxy)isophthalic acid **7** (100 mg, 1 eq) with **6a** (161 mg, 0.28 mmol, 2 equiv) using general procedure A. (*Eluent:* 1% methanol in dichloromethane) Yield: 80%, 200 mg. **^{1}H NMR (600 MHz, DMSO)** δ 10.96 (s, 2H), 10.25 (s, 2H), 8.72 (s, 1H), 8.14 (d, *J* = 9.5 Hz, 4H), 7.86 (t, *J* = 8.1 Hz, 2H), 7.59 (d, *J* = 7.9 Hz, 2H), 7.54 (d, *J* = 7.3 Hz, 4H), 7.38 (t, *J* = 7.5 Hz, 4H), 7.31 (t, *J* = 7.4 Hz, 2H), 7.15 (s, 1H), 5.50 (s, 4H); **^{13}C NMR (151 MHz, DMSO)** δ 162.63, 160.61, 156.0 (t, *J* = 26.4 Hz),), 150.77, 148.06, 140.98, 136.15, 135.50, 128.98, 128.58, 128.21, 120.4-114.7 (m), 115.69, 112.16, 112.01, 112.0-106.6 (m) 100.29, 72.08; **^{19}F NMR (565 MHz, DMSO)** -80.0 (t, *J* = 8.5 Hz),, -118.9 (q, *J* = 8.5 Hz), -126.2 (app. s); **HRMS (ESI) *m/z*:** Calcd. for $C_{40}H_{27}F_{14}N_6O_6$ $[M+H]^+$, 953.1763; Found 953.1810.

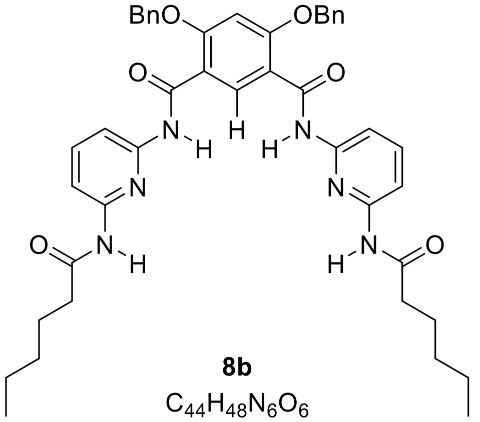

8b

$C_{44}H_{48}N_6O_6$

4,6-bis(benzyloxy)-N1,N3-bis(6-hexanamidopyridin-2-yl)isophthalamide (8b): This compound was synthesized by reacting 4,6-bis(benzyloxy)isophthalic acid **7** (100 mg, 1 eq) with **6b** (109 mg, 0.28 mmol, 2 equiv) using general procedure A. (*Eluent:* 2% mathanol in dichloromethane) Yield: 68%, 136.0 mg; **^{1}H NMR (400 MHz, DMSO)** δ 10.17 (s, 2H), 9.97 (s, 2H), 8.51 (d, *J* = 1.6 Hz, 1H), 7.92 – 7.73 (m, 6H), 7.53 (d, *J* = 7.2 Hz, 4H), 7.46 – 7.30 (m, 6H), 7.18 (s, 1H), 5.52 (s, 4H), 2.41 (t, *J* = 7.4 Hz, 4H), 1.60 (t, *J* = 7.3 Hz, 4H), 1.37 – 1.21 (m, 9H), 0.89 (t, *J* = 6.6 Hz, 6H); **^{13}C NMR (151 MHz, $CDCl_3$)** δ 206.93, 171.51, 161.71, 160.32, 149.29, 140.63, 137.80, 134.52, 128.90, 128.72, 115.02,

109.44, 109.01, 97.77, 72.12, 37.71, 31.56, 30.93, 24.99, 22.47, 13.97.**HRMS (ESI) *m/z*:** Calcd. for $C_{44}H_{49}N_6O_6$ $[M+H]^+$, 757.3708; Found 757.3744.

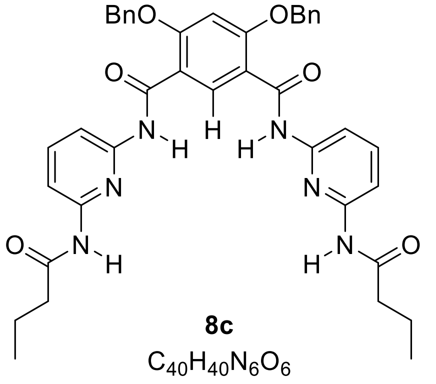

8c
$C_{40}H_{40}N_6O_6$

4,6-bis(benzyloxy)-N1,N3-bis(6-butyramidopyridin-2-yl)isophthalamide (8c): This compound was synthesized by reacting 4,6-bis(benzyloxy)isophthalic acid **7** (100 mg, 1 eq) with **6c** (94 mg, 0.28 mmol, 2 eq) using general procedure A. (*Eluent:* 2% methanol in dichloromethane) Yield: 75%, 138 mg; ^{1}H NMR (600 MHz, DMSO) δ 10.21 (s, 2H), 9.77 (s, 2H), 8.68 (s, 1H), 8.09 (d, *J* = 3.8 Hz, 2H), 7.83 (d, *J* = 7.2 Hz, 2H), 7.73 (q, *J* = 6.0 Hz, 4H), 7.49 (d, *J* = 7.3 Hz, 4H), 7.38 (t, *J* = 7.5 Hz, 4H), 7.35 – 7.30 (m, 2H), 7.05 (d, *J* = 2.5 Hz, 1H), 5.47 (s, 4H), 2.38 (t, *J* = 7.3 Hz, 4H), 1.65 (h, *J* = 7.4 Hz, 4H), 0.95 (t, *J* = 7.4 Hz, 6H); **^{13}C NMR (151 MHz, DMSO)** δ 172.55, 162.68, 160.38, 150.35, 149.70, 140.84, 136.05, 135.55, 129.06, 128.59, 127.95, 115.90, 109.66, 109.33, 100.28, 71.85, 38.70, 18.80, 13.96; **HRMS (ESI) *m/z*:** Calcd. for $C_{40}H_{40}N_6O_6Na$ $[M+Na]^+$, 723.2902; Found 723.2933.

General procedure B: synthesis of compounds 1-3:

In a 25 mL round bottom flask, **8a-8c** (100 mg) were dissolved in THF:methanol (3:1, 50 mL) to which 10% Pd/C (0.02 mg) was added. The reaction was subjected to stirring for a duration of 3 h under a hydrogen gas atmosphere (1 atm). Following the reaction, the Pd/C was filtered out, and the resulting filtrate was collected and dried under vacuum to yield a crude product that was purified via column chromatography to furnish compounds **1-3** in excellent yields.

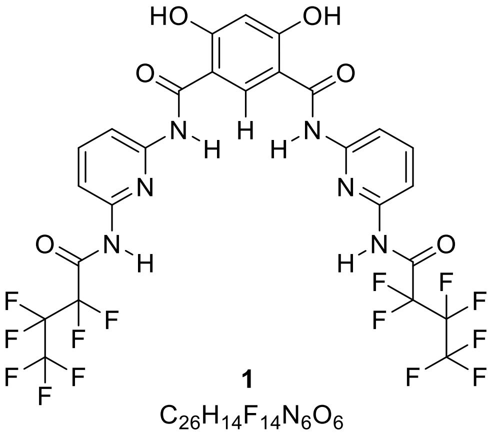

1
$C_{26}H_{14}F_{14}N_6O_6$

N1,N3-bis(6-(2,2,3,3,4,4,4-heptafluorobutanamido)pyridin-2-yl)-4,6-dihydroxyisophthalamide (**1**): The crude product was purified by column chromatography over silica gel (*Eluent*: 1% methanol in dichloromethane) to furnish **1** as a white solid (77.0 mg, 95%); **^{1}H NMR (400 MHz, DMSO)** δ 12.42 (s, 2H), 11.97 (s, 2H), 10.62 (s, 2H), 8.81 (s, 1H), 8.18 (d, *J* = 8.2 Hz, 2H), 7.96 (t, *J* = 8.1 Hz, 2H), 7.64 (d, *J* = 8.0 Hz, 2H), 6.70 (s, 1H); **^{13}C NMR (151 MHz, DMSO)** δ 163.50, 161.31, 156.3 (t, *J* = 26.4 Hz), 150.98, 148.52, 141.50, 136.64, 120.4-114.7

(m), 112.23, 112.05, 111.9-106.6 (m), 104.15; **^{19}F NMR (565 MHz, DMSO)** δ -79.99 (t, *J* = 8.5 Hz), -118.56 (q, *J* = 8.5 Hz), -126.23. **HRMS (ESI) *m/z*:** Calcd. for $C_{26}H_{15}F_{14}N_6O_6$ [M+H]$^+$, 773.0824; Found 773.0811.

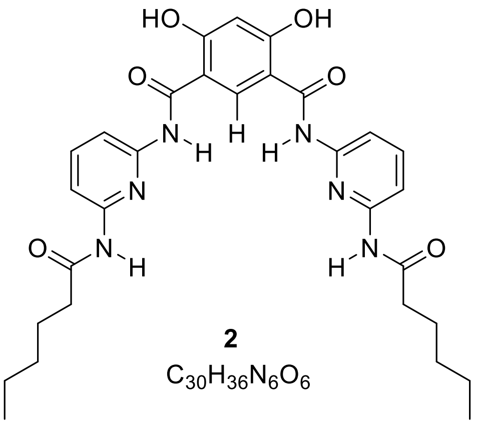

2

$C_{30}H_{36}N_6O_6$

N1,N3-bis(6-hexanamidopyridin-2-yl)-4,6-dihydroxyisophthalamide (**2**): The crude product was purified by column chromatography over silica gel (*Eluent*: 5% methanol in dichloromethane) to furnish **2** as a white solid (70 mg, 92%); **^{1}H NMR (400 MHz, DMSO)** δ 12.36 (s, 2H), 10.57 (s, 2H), 10.33 (s, 2H), 8.81 (s, 1H), 7.98 (d, *J* = 7.7 Hz, 2H), 7.80 (dt, *J* = 15.9, 8.0 Hz, 4H), 6.67 (s, 1H), 2.37 (t, *J* = 7.4 Hz, 4H), 1.57 (p, *J* = 7.2 Hz, 4H), 1.27 (dp, *J* = 15.6, 6.8 Hz, 8H), 0.86 (t, *J* = 6.7 Hz, 6H); **^{13}C NMR (151 MHz, DMSO)** δ 172.77, 163.41, 151.22, 150.58, 140.79, 136.71, 128.90, 127.61, 109.45, 109.40, 104.21, 36.48, 31.31, 25.10, 22.36, 14.32; **HRMS (ESI) *m/z*:** Calcd. for $C_{30}H_{37}N_6O_6$ [M+H]$^+$, 577.2769; Found 577.2763.

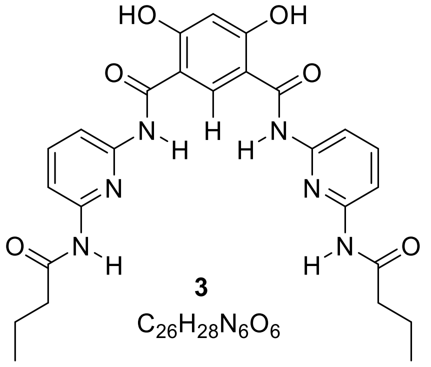

3

$C_{26}H_{28}N_6O_6$

N1,N3-bis(6-butyramidopyridin-2-yl)-4,6-dihydroxyisophthalamide (**3**): The crude product was purified by column chromatography over silica gel (*Eluent*: 2% methanol in dichloromethane) to furnish **3** as a white solid (67 mg, 90%); **^{1}H NMR (600 MHz, DMSO)** δ 12.31 (s, 2H), 10.43 (s, 2H), 10.33 (s, 2H), 8.81 (s, 1H), 7.97 (dd, *J* = 7.9, 0.9 Hz, 2H), 7.85 (d, *J* = 8.0 Hz, 2H), 7.80 (t, *J* = 8.0 Hz, 2H), 6.71 (s, 1H), 2.37 (t, *J* = 7.3 Hz, 4H), 1.61 (h, *J* = 7.4 Hz, 4H), 0.91 (t, *J* = 7.4 Hz, 6H); **^{13}C NMR (151 MHz, DMSO)** δ 172.61, 163.20, 160.92, 151.21, 150.44, 140.84, 136.70, 112.27, 109.60, 109.45, 104.12, 38.43, 18.84, 14.07. **HRMS (ESI) *m/z*:** Calcd. for $C_{26}H_{29}N_6O_6$ [M+H]$^+$, 521.2143; Found 521.2141.

Synthesis of photocaged compound 1b:

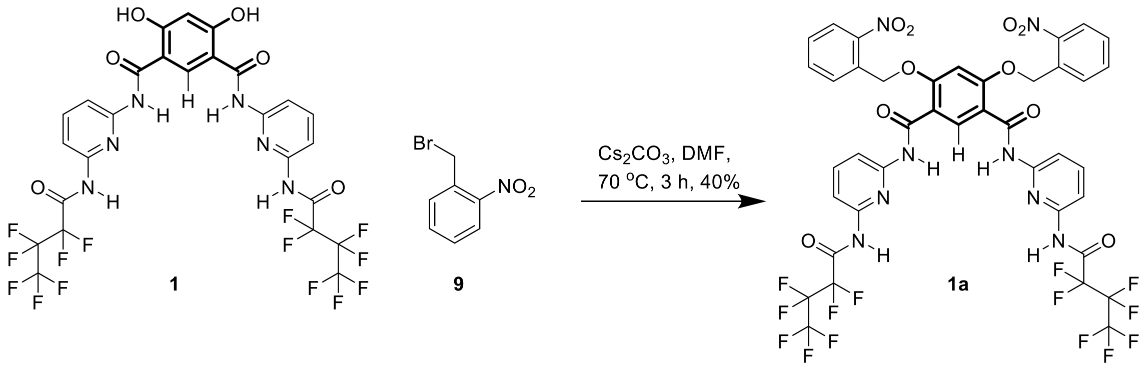

Scheme 2. Chemical synthesis of compounds **1b**.

N1,N3-bis(6-(2,2,3,3,4,4,4-heptafluorobutanamido)pyridin-2-yl)-4,6-bis((2-nitrobenzyl)oxy)isophthalamide (1a): In a 50 mL round bottomed flask, **1** (100 mg, 0.129 mmol, 1 equiv) and Cs_2CO_3 (92.0 mg, 0.284 mmol, 2.2 equiv) were dissolved in DMF (10 mL), followed by the addition 1-(bromomethyl)-2-nitrobenzene **9** (61.0 mg, 0.284 mmol, 2.2 equiv). The reaction mixture was stirred for 3 h at 80 °C using an oil bath. After completion, water (20 mL) was added to the reaction mixture, and extracted with ethyl acetate (3 × 30 mL). The organic layers were dried with anhydrous magnesium sulfate, filtered, and concentrated under reduced pressure to give the crude product. The crude product was purified by column chromatography over silica gel (*Eluent*: 2 to 5% methanol in dichloromethane) to furnish **4a** as a white solid (46.0 mg, 40%); **^{1}H NMR (400 MHz, DMSO)** δ 11.59 (s, 2H), 10.34 (s, 2H), 8.43 (s, 1H), 8.16 – 8.09 (m, 4H), 7.98 (t, J = 8.1 Hz, 2H), 7.73 (d, J = 7.7 Hz, 2H), 7.69 – 7.62 (m, 2H), 7.57 (t, J = 8.1 Hz, 4H), 6.89 (s, 1H), 5.80 (s, 4H); **^{13}C NMR (151 MHz, DMSO)** δ 163.46, 159.81, 156.3-156.0 (m), 150.99, 148.14, 147.45, 141.41, 134.68, 131.54, 129.79, 129.55, 129.06, 125.56, 120.5-114.7 (m), 116.86, 112.85, 112.53, 110.3-106.4 (m), 100.31, 68.60; **^{19}F NMR (565 MHz, DMSO)** δ -79.98 (t, J = 8.4 Hz), -118.70 (m), -126.24 (app. s); **HRMS (ESI) *m/z*:** Calcd. for $C_{40}H_{25}F_{14}N_8O_{10}$ $[M+H]^+$, 1043.1465; Found 1043.1444.

III. NMR Spectra

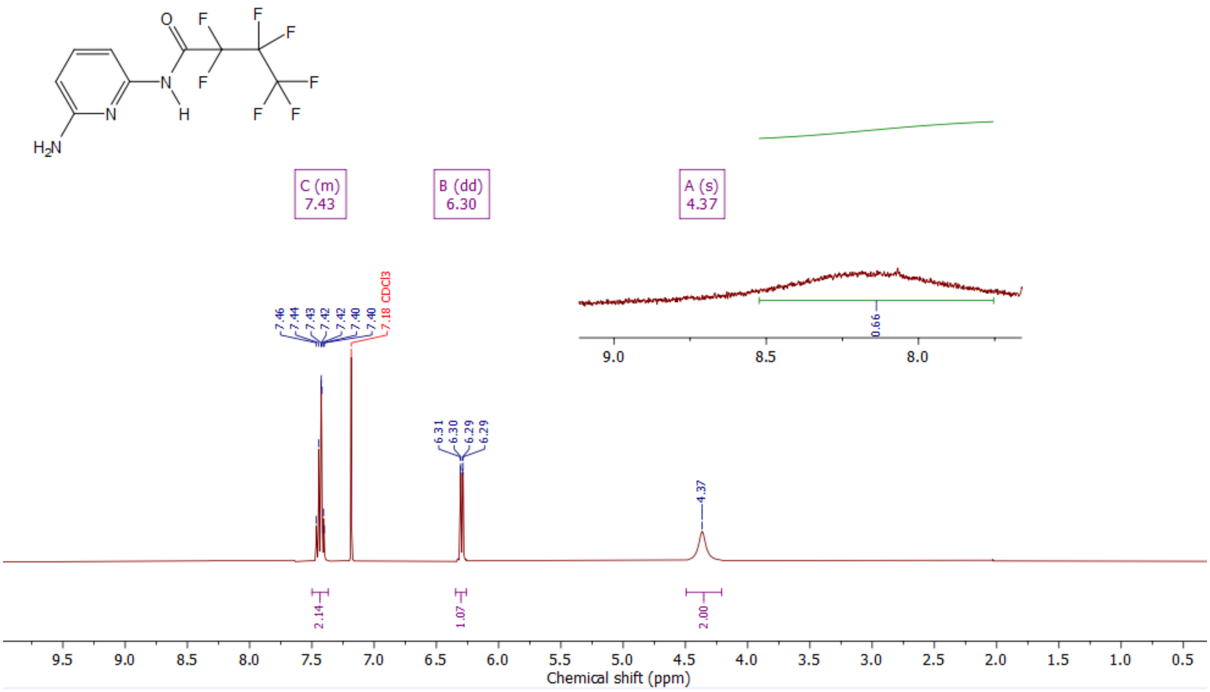

Figure S1: ^{1}H NMR spectrum of **6a** in $CDCl_3$ (600 MHz, 298 K).

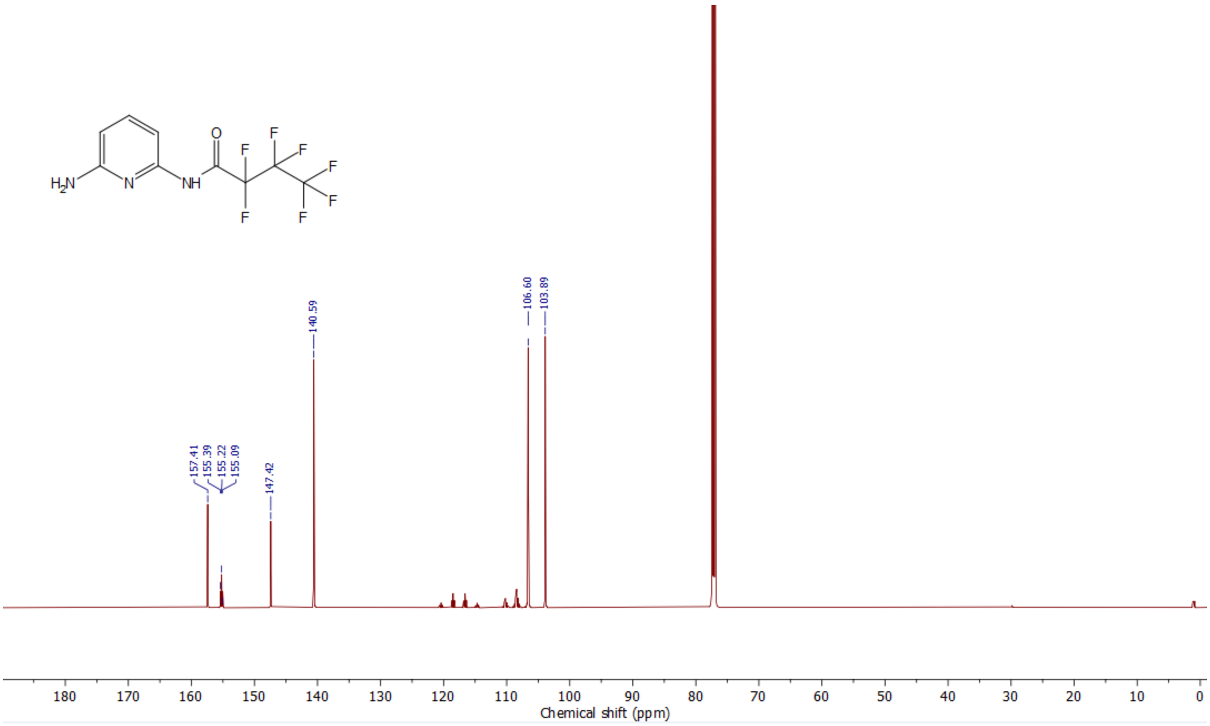

Figure S2: ^{13}C NMR spectrum of **6a** in $CDCl_3$ (151 MHz, 298 K).

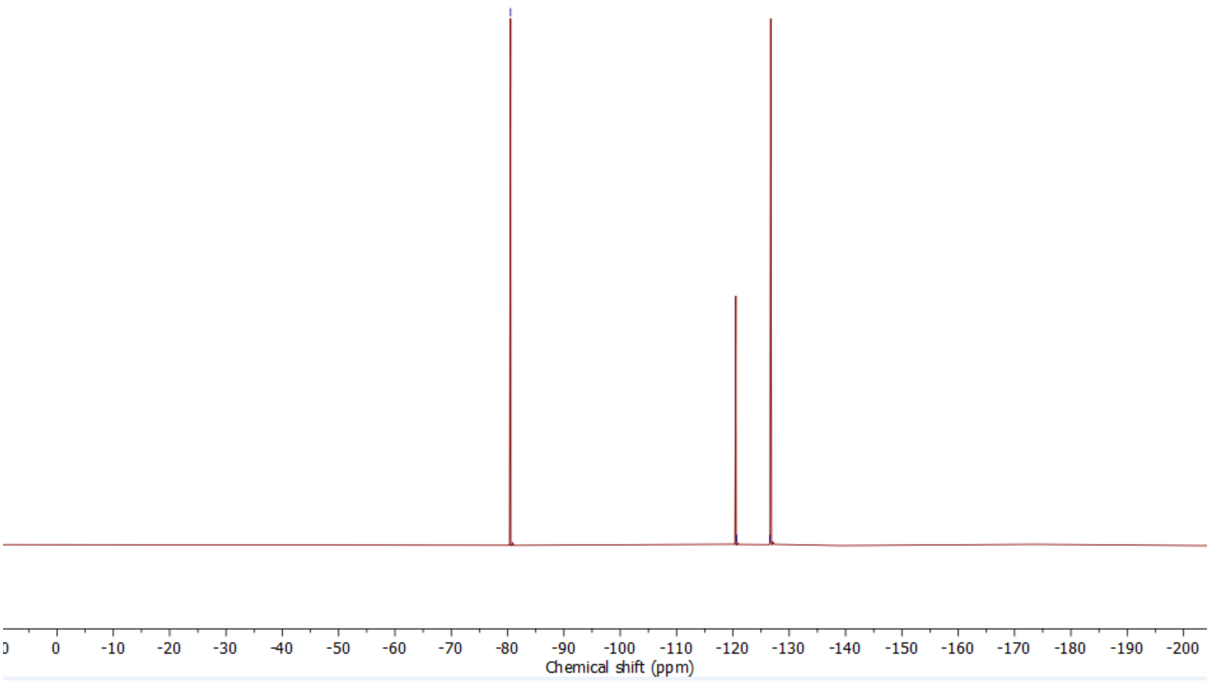

Figure S3: ^{19}F NMR spectrum of **6a** in $CDCl_3$ (565 MHz, 298 K).

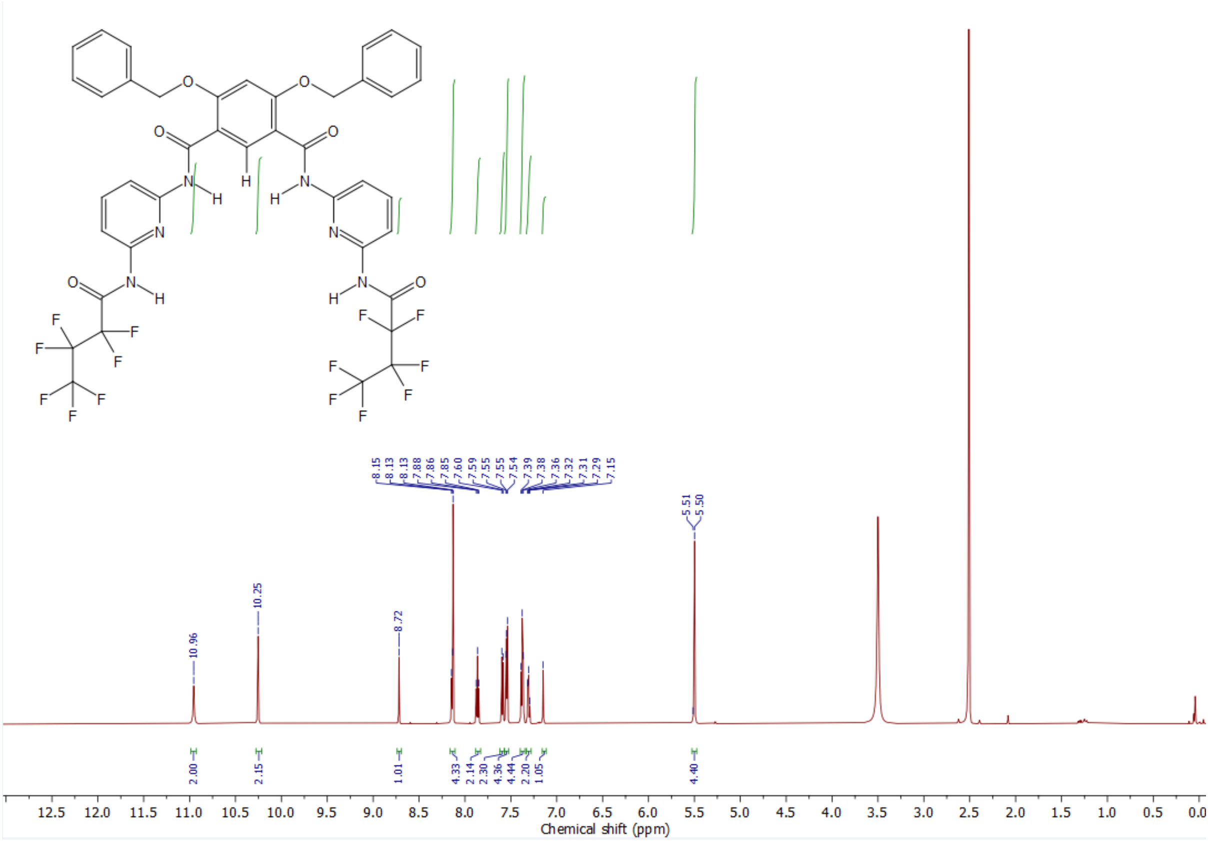

Figure S4: ^{1}H NMR spectrum of **8a** in DMSO-d_6 (600 MHz, 298 K).

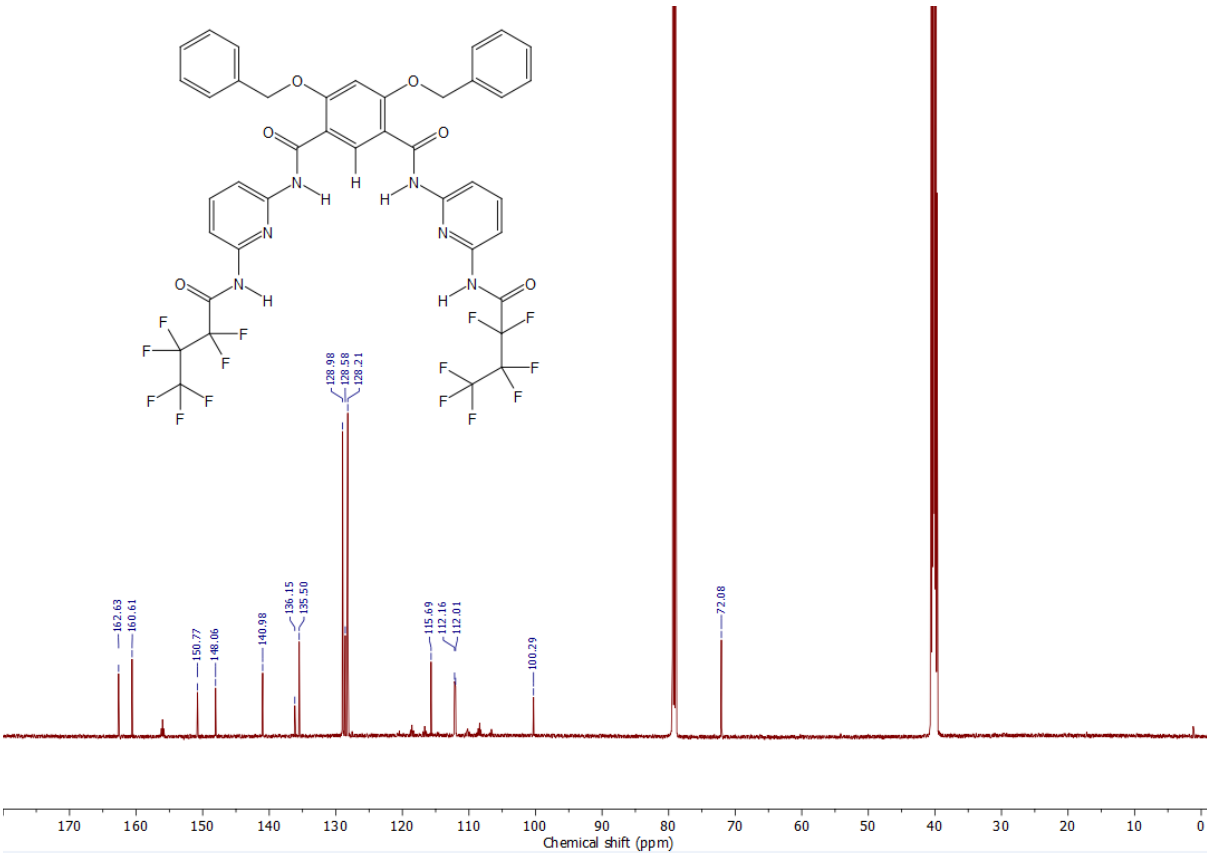

Figure S5: ^{13}C NMR spectrum of **8a** in 1:1 $CDCl_3$ / DMSO-d_6 (151, MHz, 298 K).

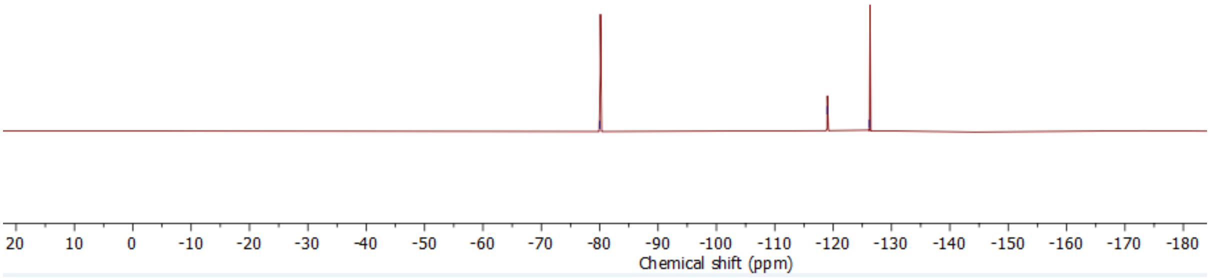

Figure S6: ^{19}F NMR spectrum of **8a** in 3:1 $CDCl_3$ / DMSO-d_6 (565 MHz, 298 K).

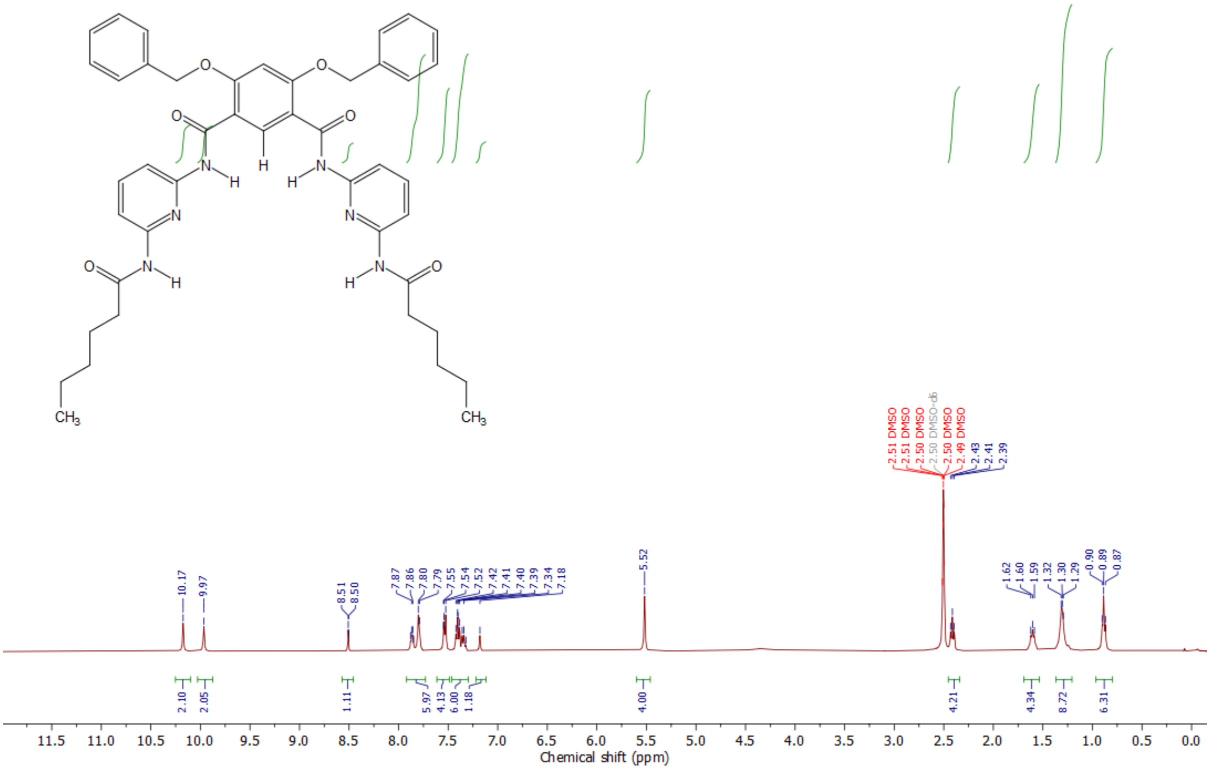

Figure S7: ¹H NMR spectrum of **8b** in DMSO-d_6 (600, MHz, 298 K).

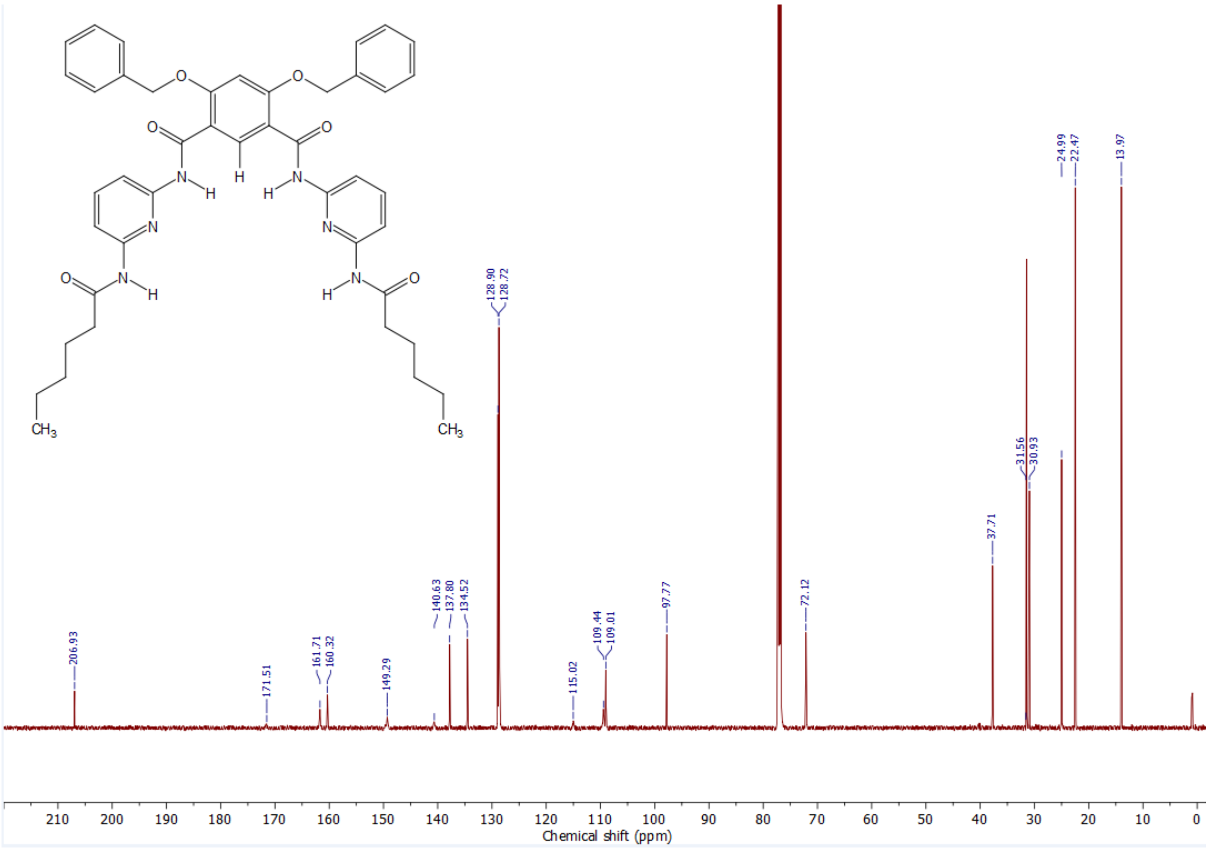

Figure S8: ¹³C NMR spectrum of **8b** in $CDCl_3$ (151 MHz, 298 K).

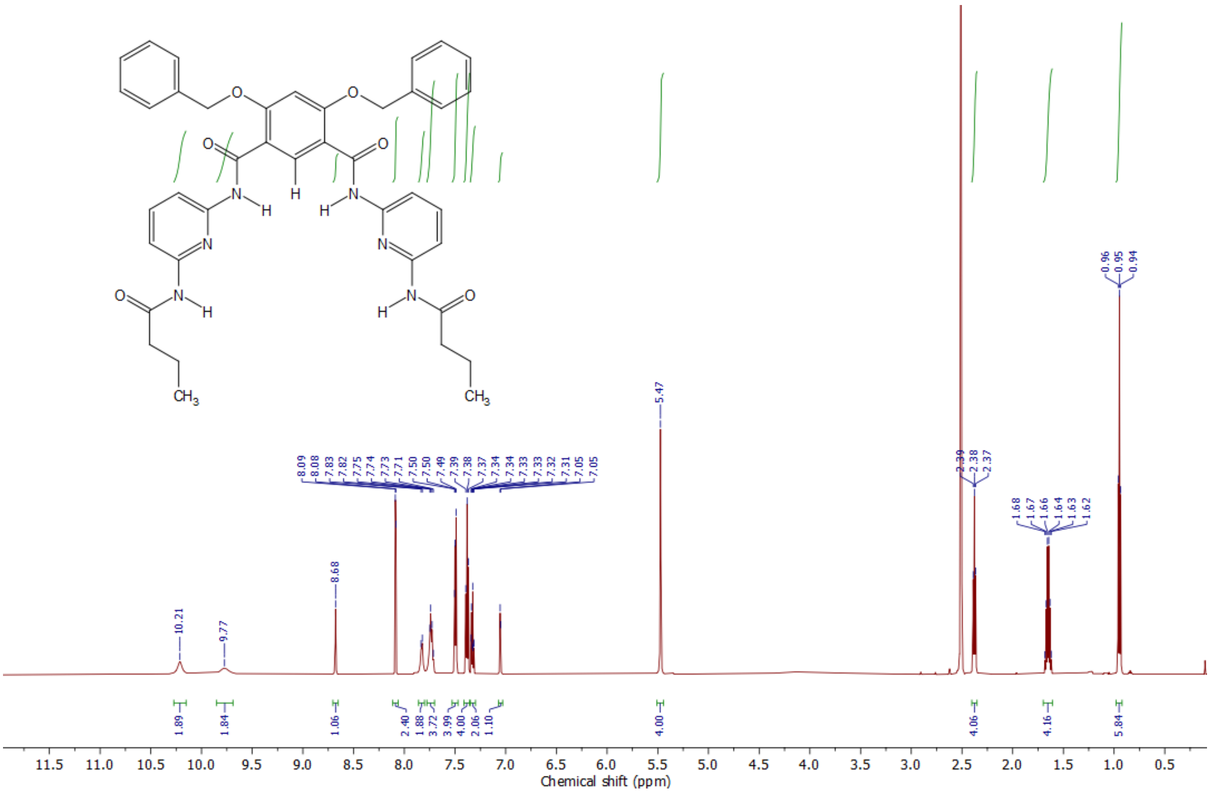

Figure S9: ^{1}H NMR spectrum of **8c** in DMSO-d_6 (600 MHz, 298 K).

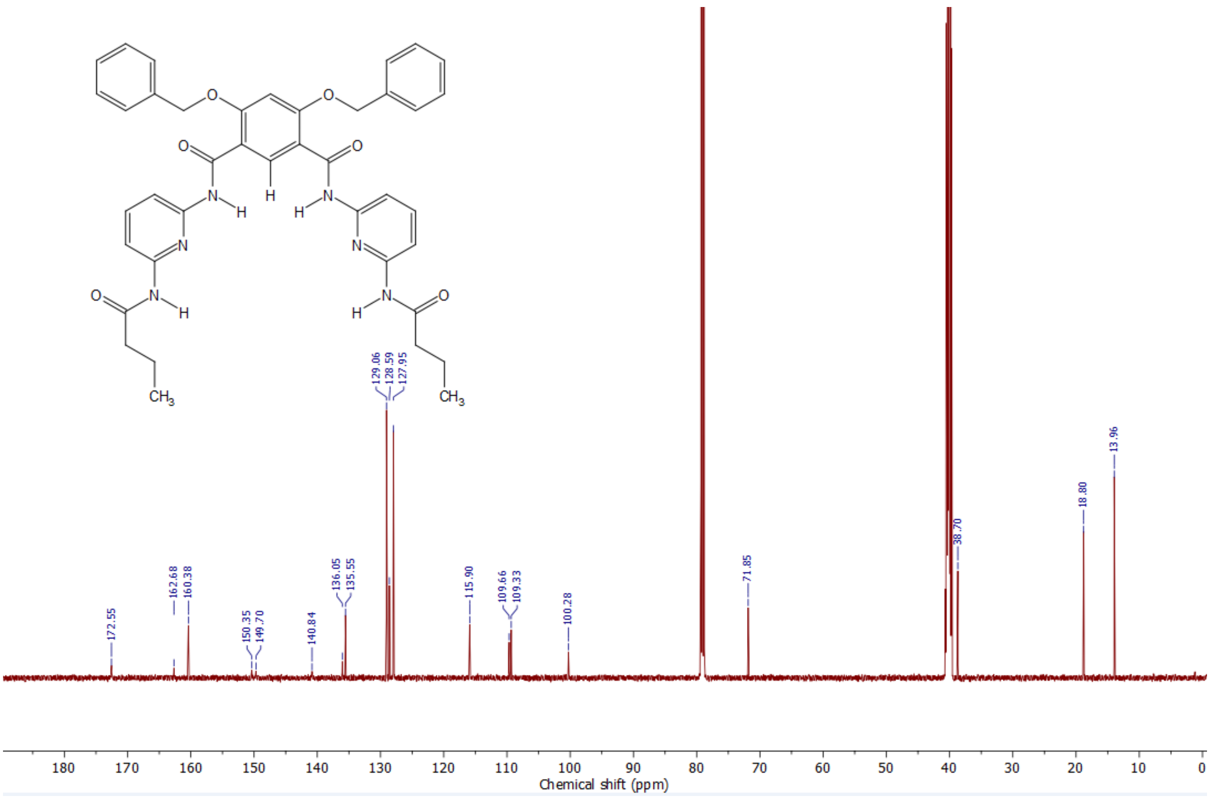

Figure S10: ^{13}C NMR spectrum of **8c** in 1:1 $CDCl_3$ / DMSO-d_6 (151 MHz, 298 K)..

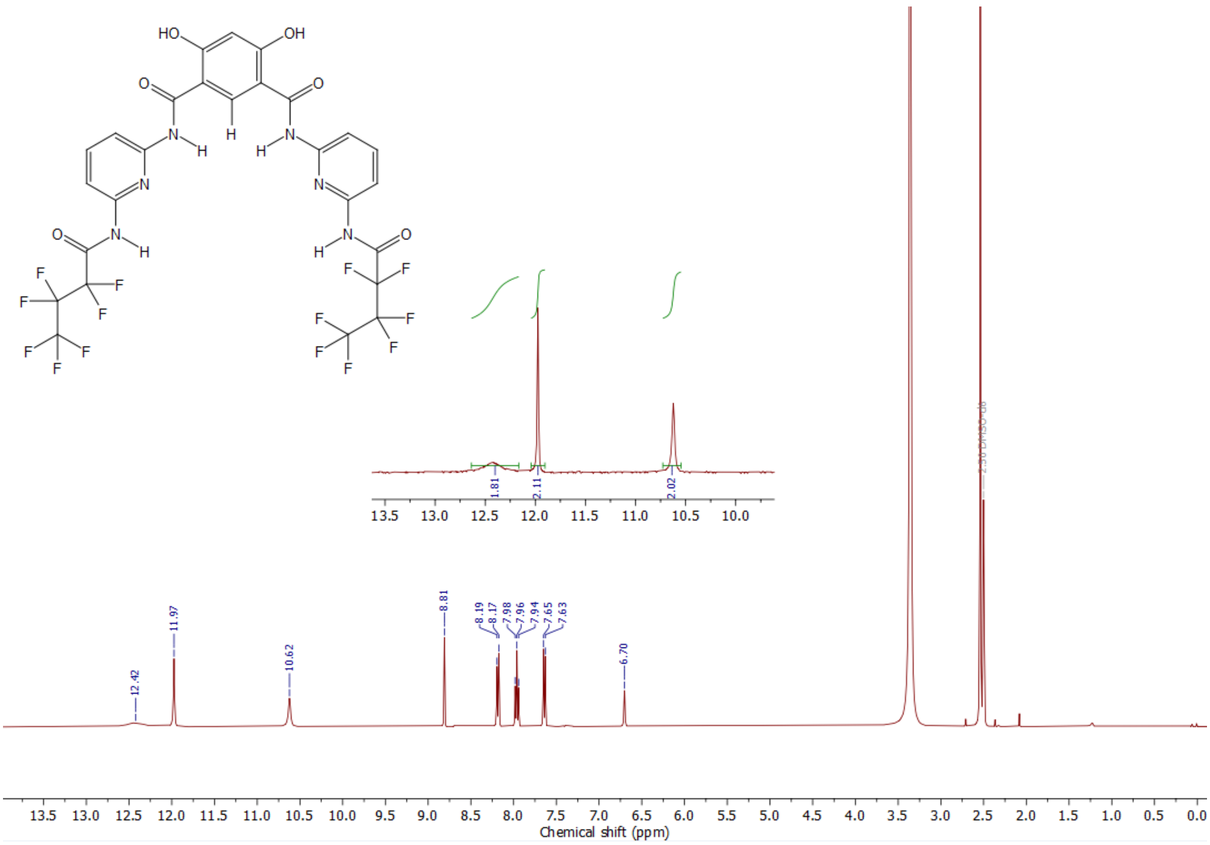

Figure S11: ^{1}H NMR spectrum of **1** in DMSO-d_6 (600 MHz, 298 K).

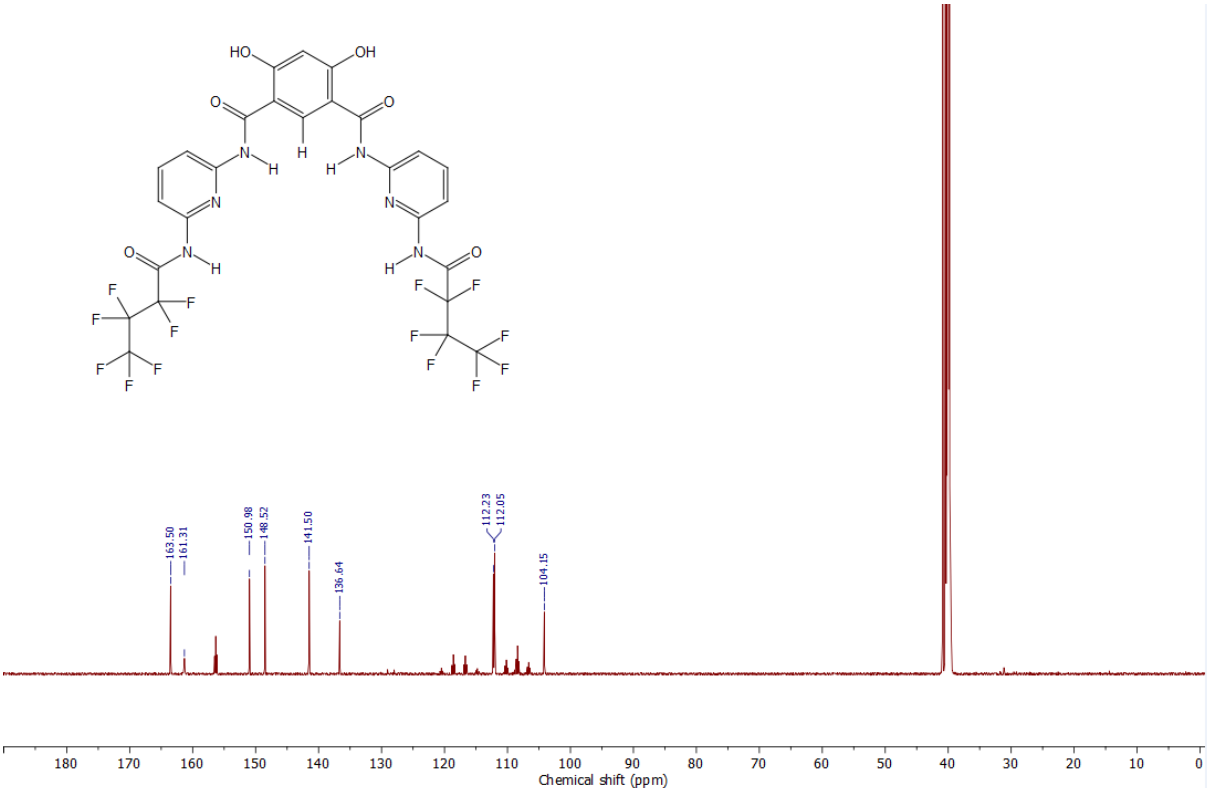

Figure S12: ^{13}C NMR spectrum of **1** in DMSO-d_6 (151 MHz, 298 K).

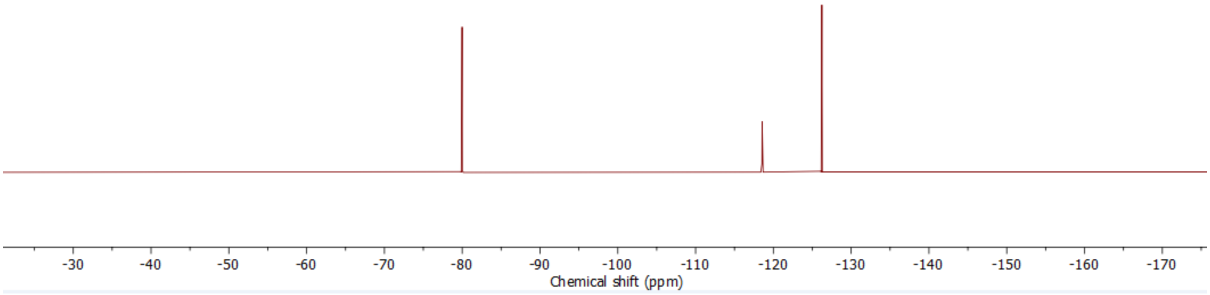

Figure S13: ^{19}F NMR spectrum of **1** in DMSO-d_6 (565 MHz, 298 K).

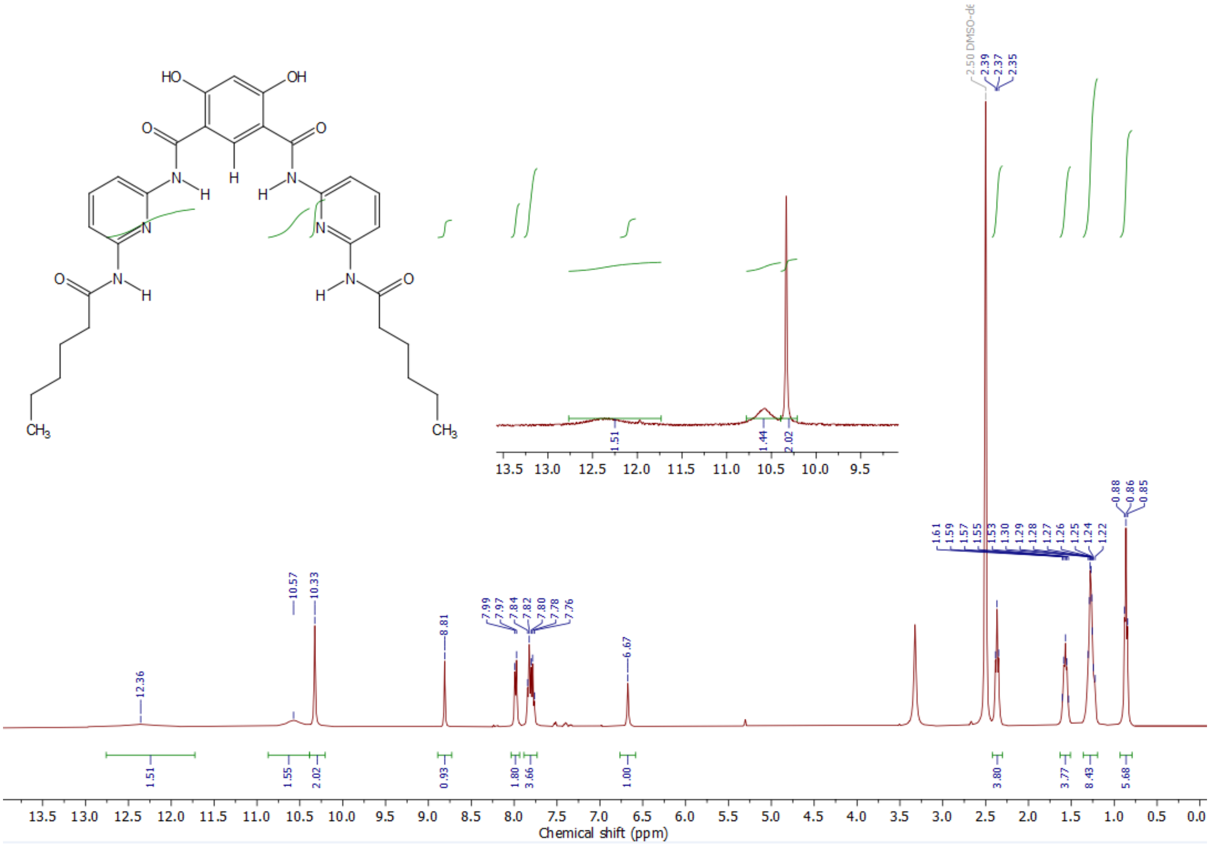

Figure S14: ^{1}H NMR spectrum of **2** in DMSO-d_6 (600 MHz, 298 K).

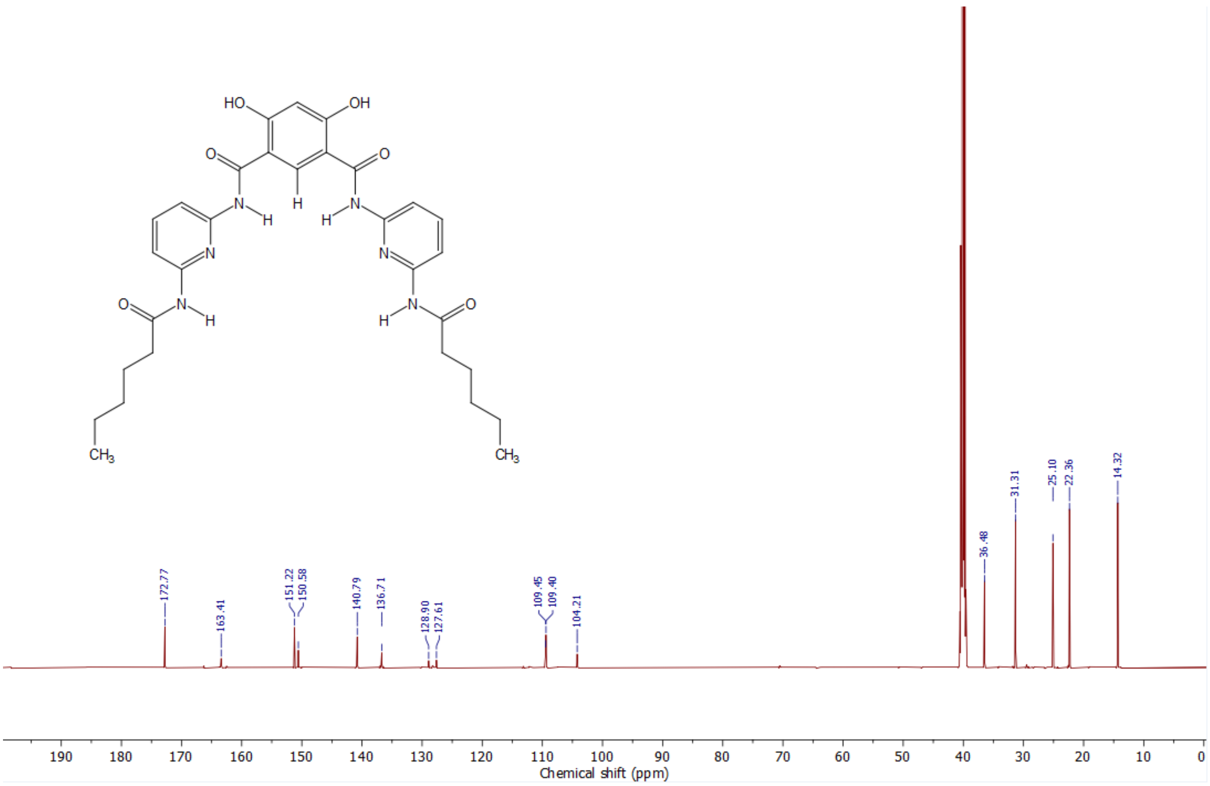

Figure S15: ^{13}C NMR spectrum of **2** in DMSO-d_6 (151 MHz, 298 K).

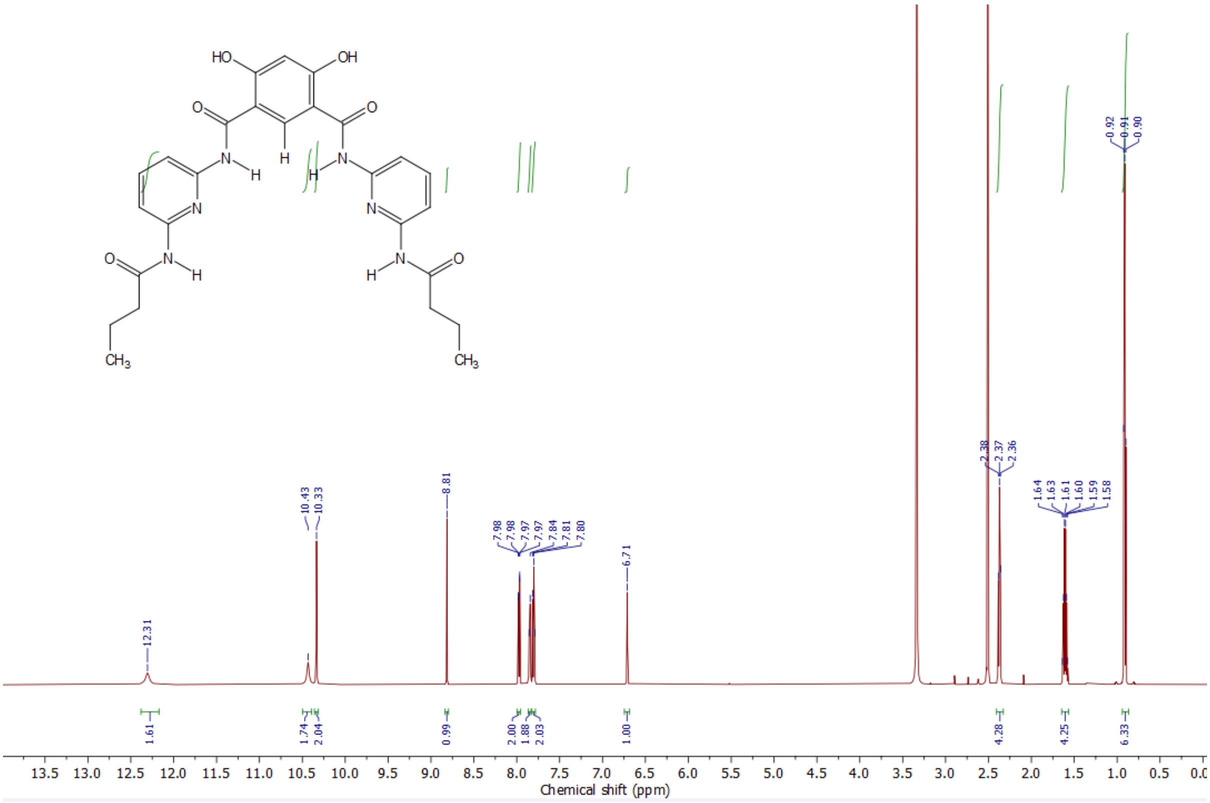

Figure S16: ^{1}H NMR spectrum of **3** in DMSO-d_6 (600 MHz, 298 K).

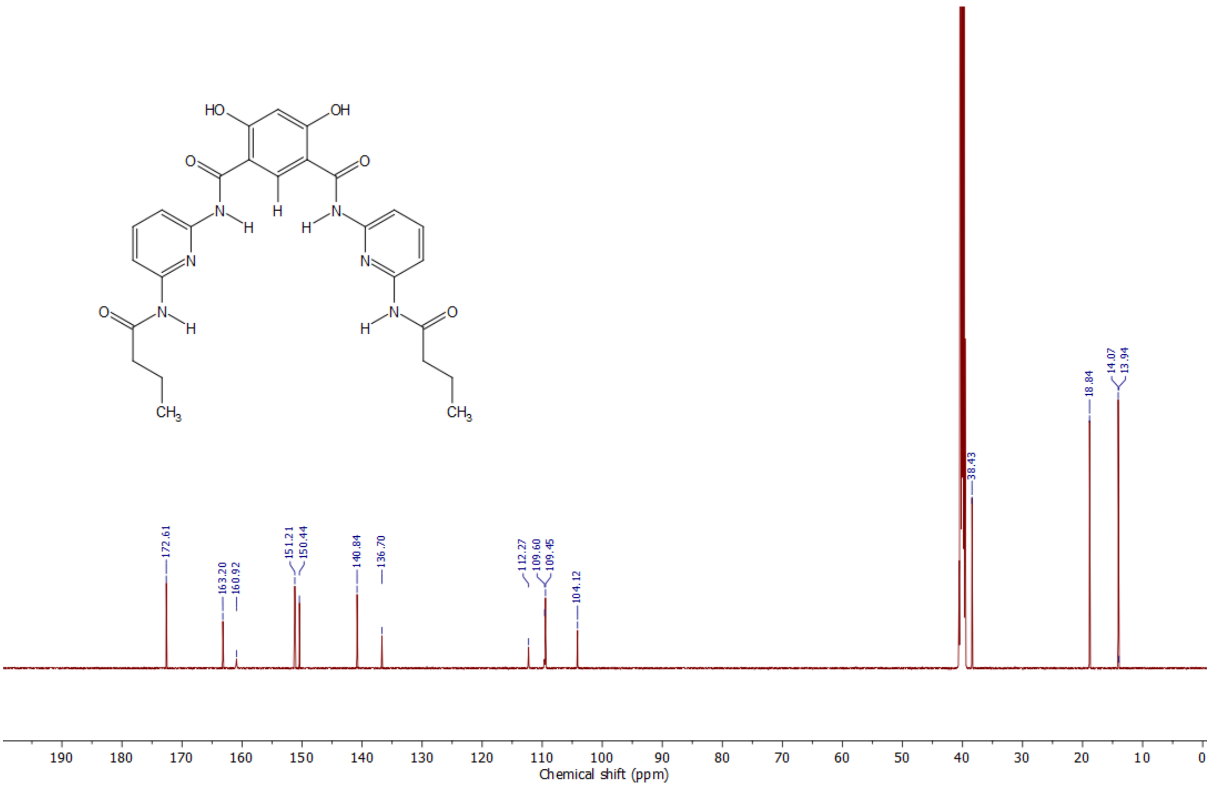

Figure S17: ^{13}C NMR spectrum of **3** in DMSO-d_6 (151 MHz, 298 K).

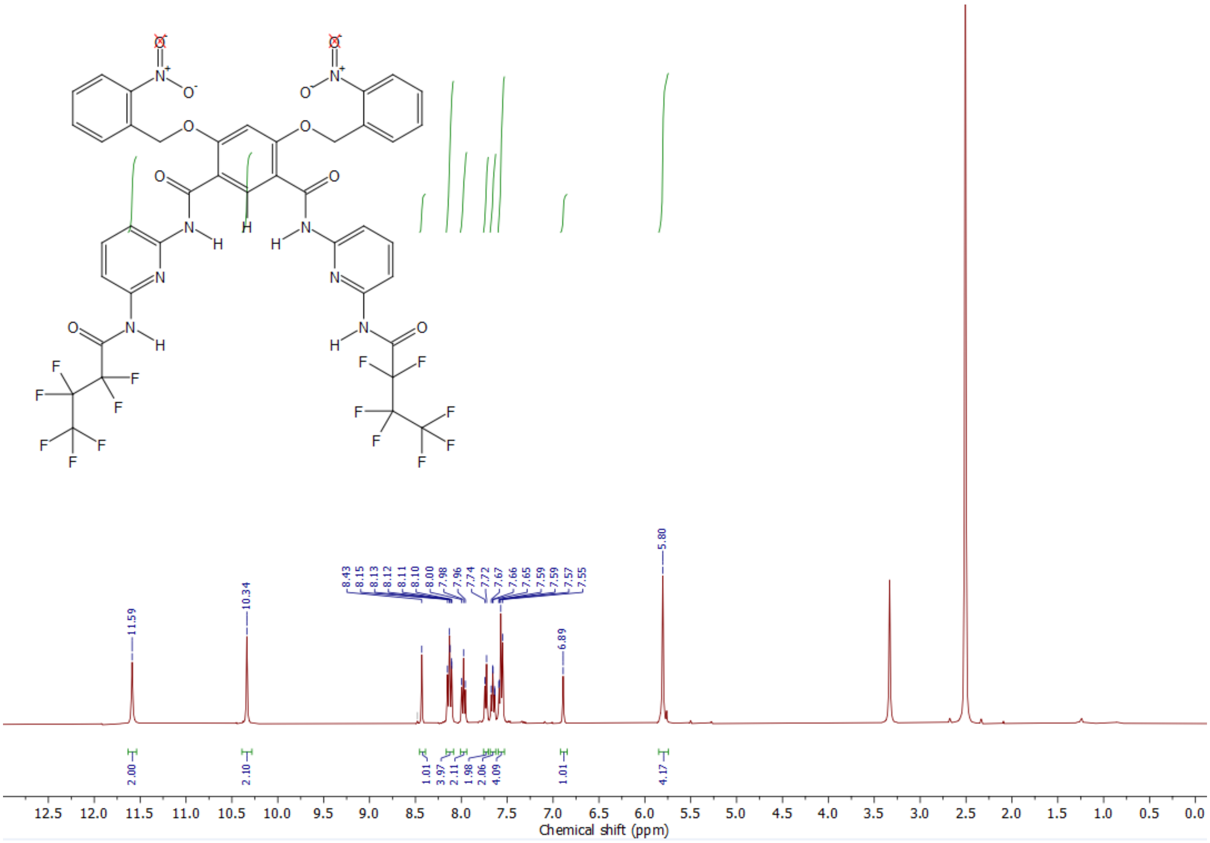

Figure S18: ^{1}H NMR spectrum of **1a** in DMSO-d_6 (600 MHz, 298 K).

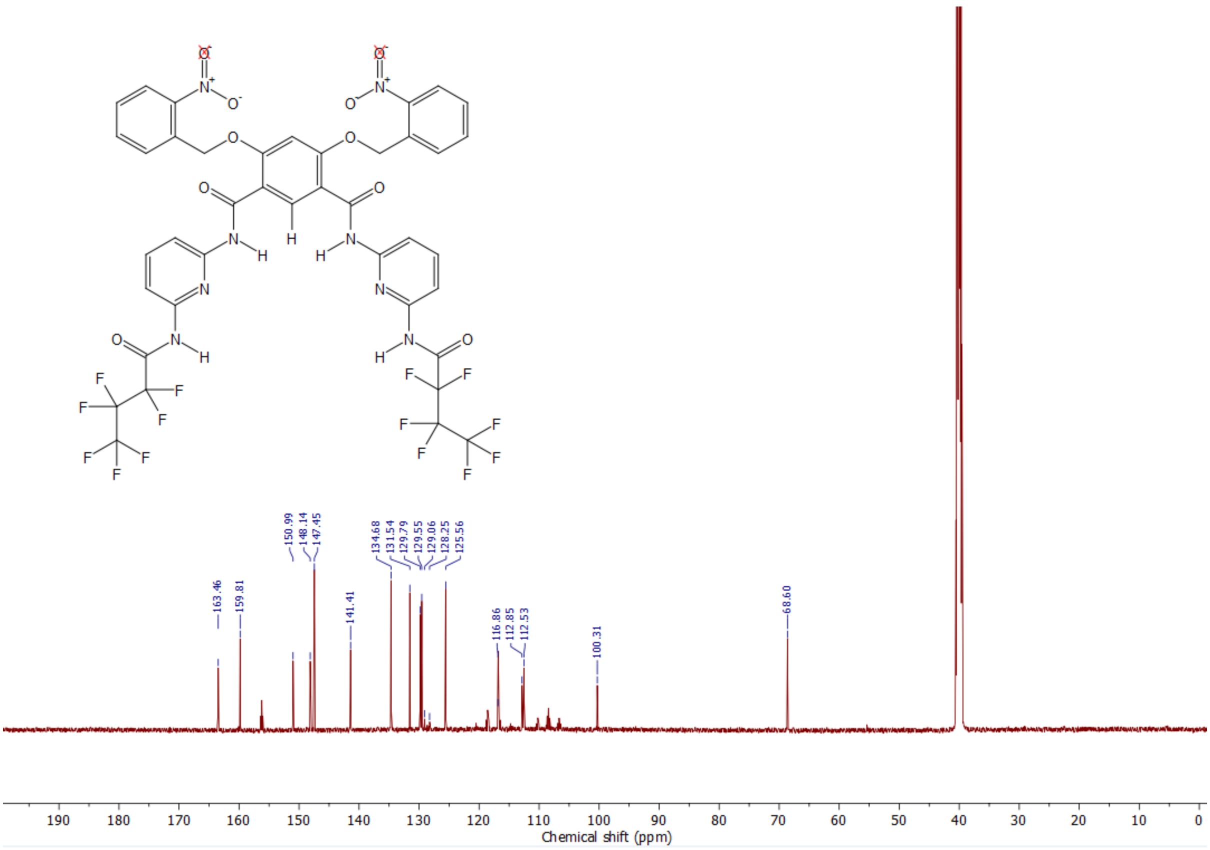

Figure S19: ^{13}C NMR spectrum of **1a** in DMSO-d_6 (151 MHz, 298 K).

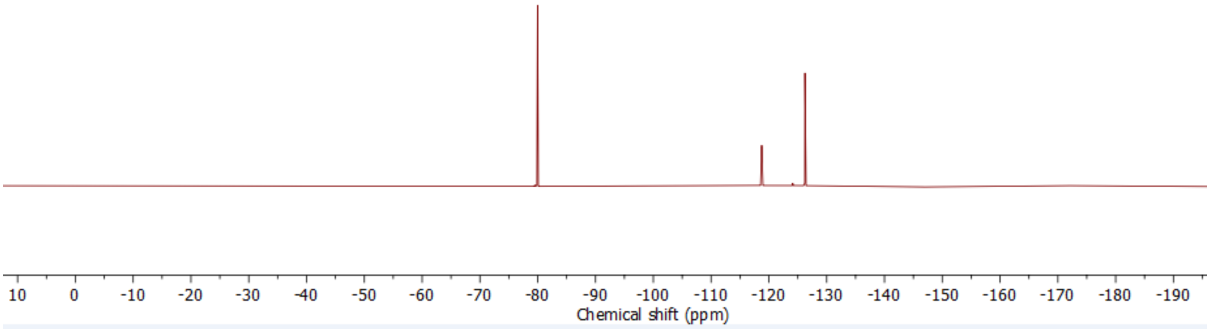

Figure S20: ^{19}F NMR Spectrum of **1a** in DMSO-d_6 (565 MHz, 298 K).

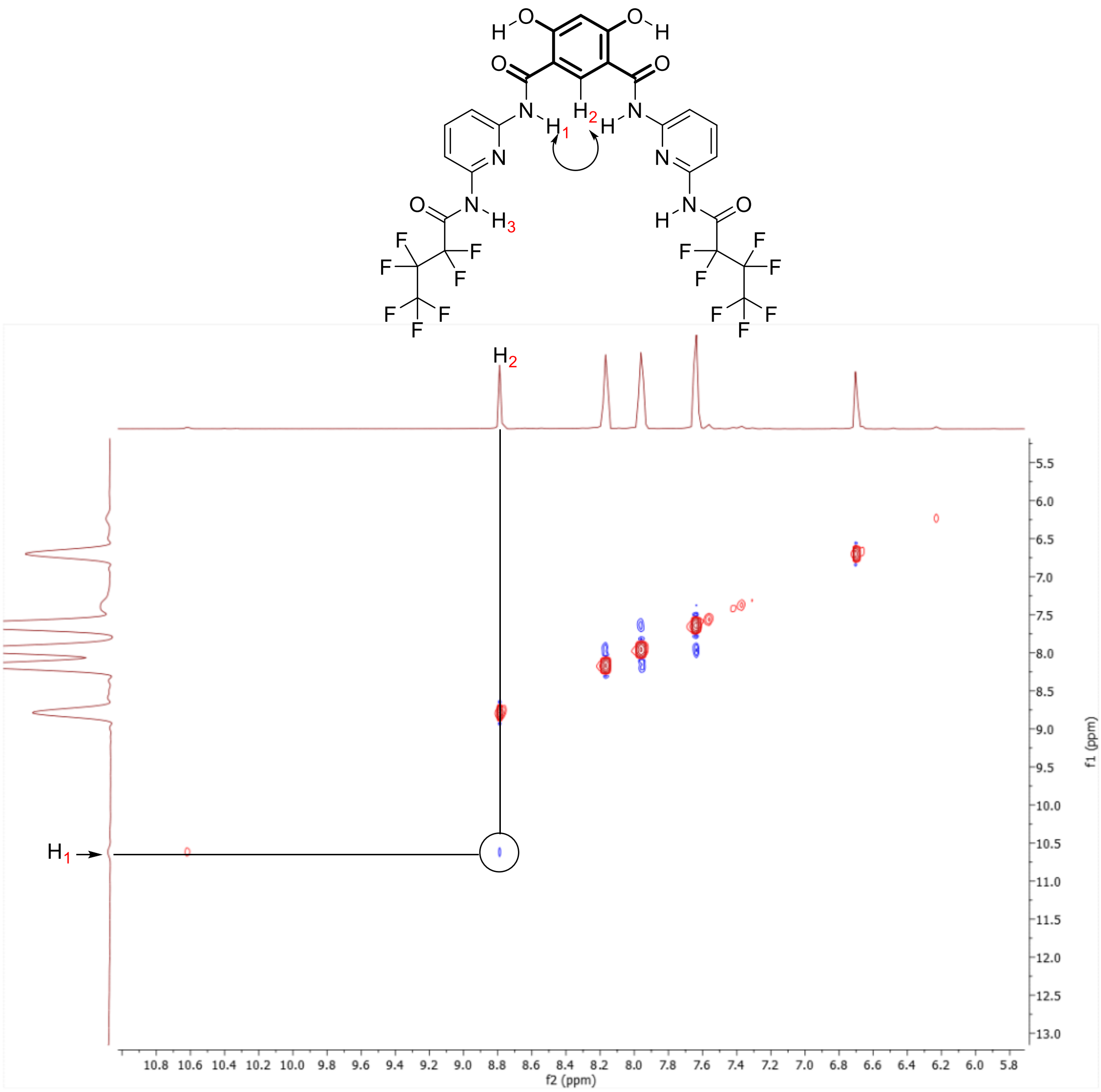

Figure S21. ROESY NMR of **1** in DMSO-d_6.

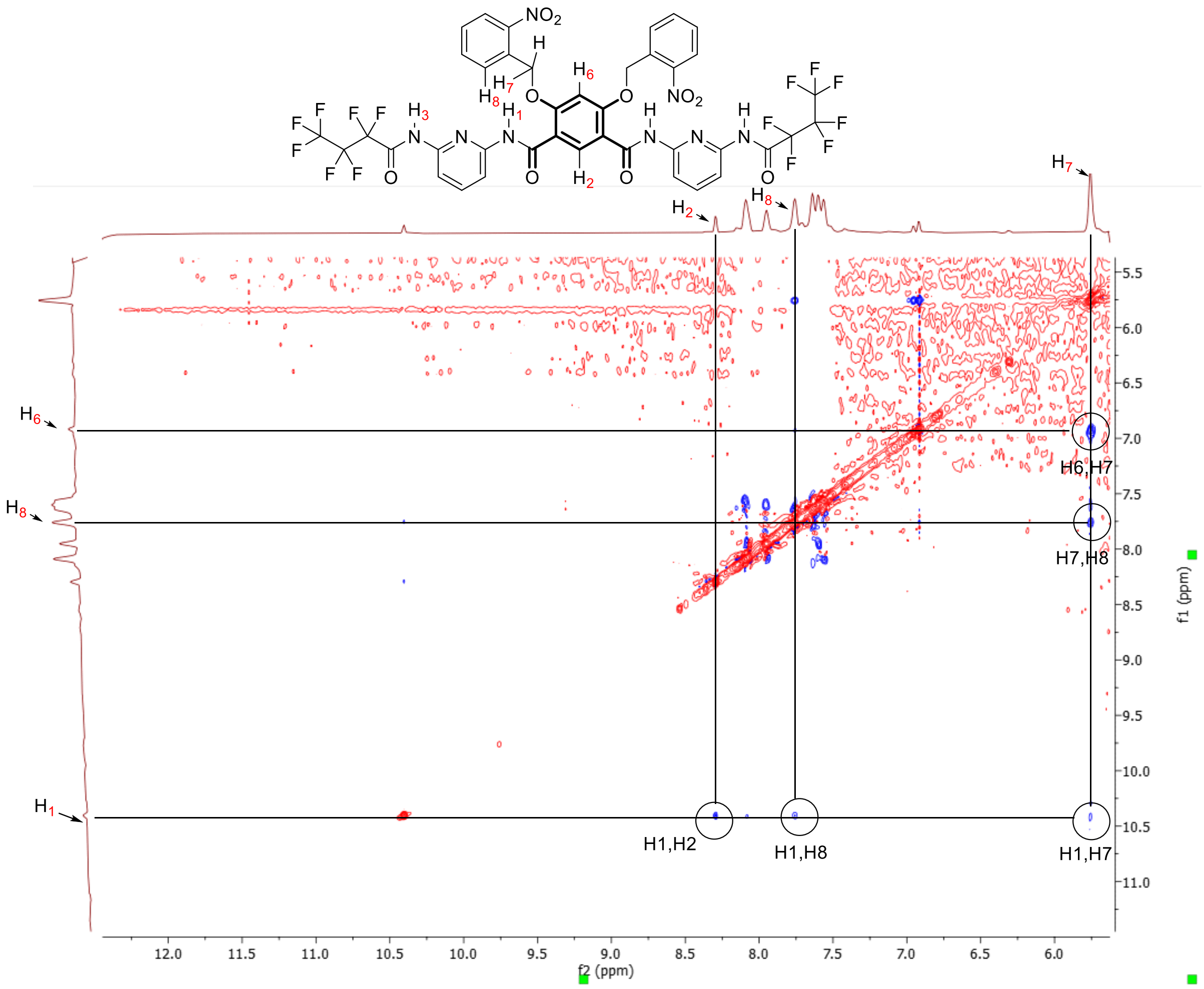

Figure S22. ROESY NMR of **1a** in DMSO-d_6.

IV. Anion Binding Studies

^{1}H NMR titration experiments were carried out at 298K on a Bruker 400 MHz spectrometer. The residual solvent signal (THF-d_8, $\delta_H = 3.58$) was used as an internal reference to calibrate the spectra. TBACl, $TBANO_3$ and receptor were dried under a high vacuum before use. The titrations were performed by the addition of either TBACl or $TBANO_3$ (0.1 M in THF-d_8) to **1** and TBACl to **1a** (1.0 mM), respectively. All NMR data were processed using MestReNova 6.0 and the collected data analysed using BindFit v0.5.[3]

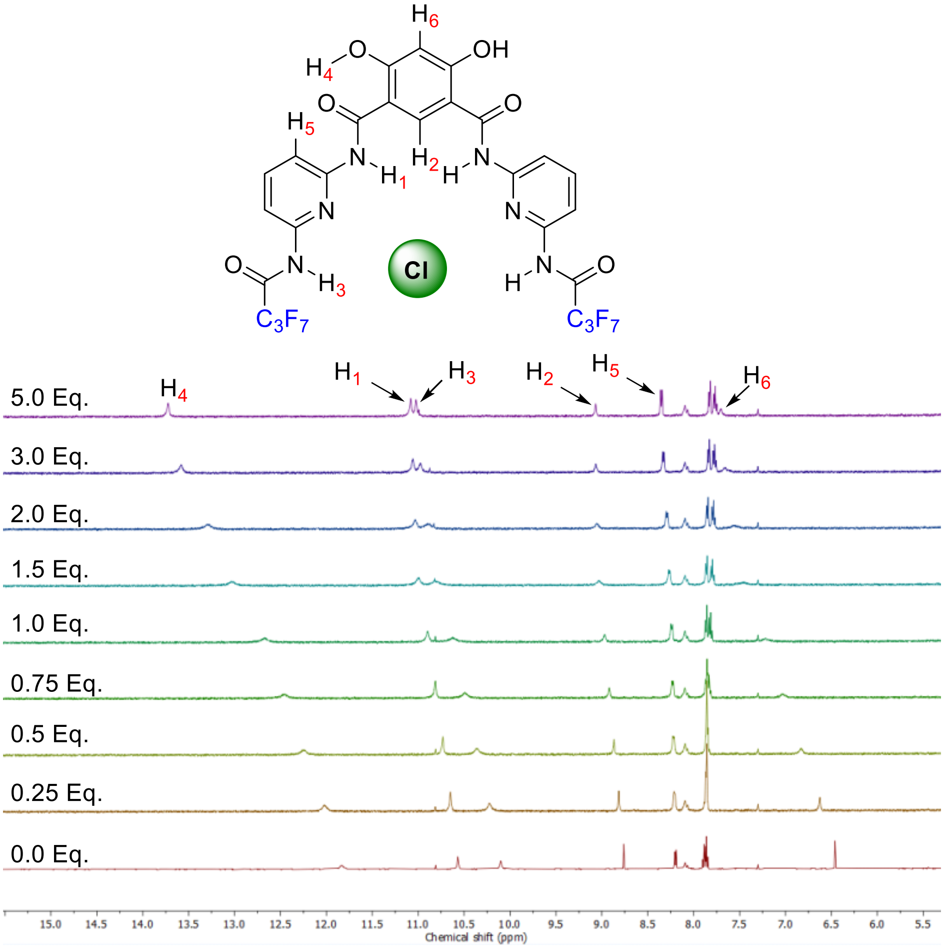

Figure S23. ^{1}H NMR titration spectra for **1** (1.0 mM in THF-d_8) with stepwise addition of TBACl in THF-d_8. The equivalents of added TBACl are reported on the stacked spectra.

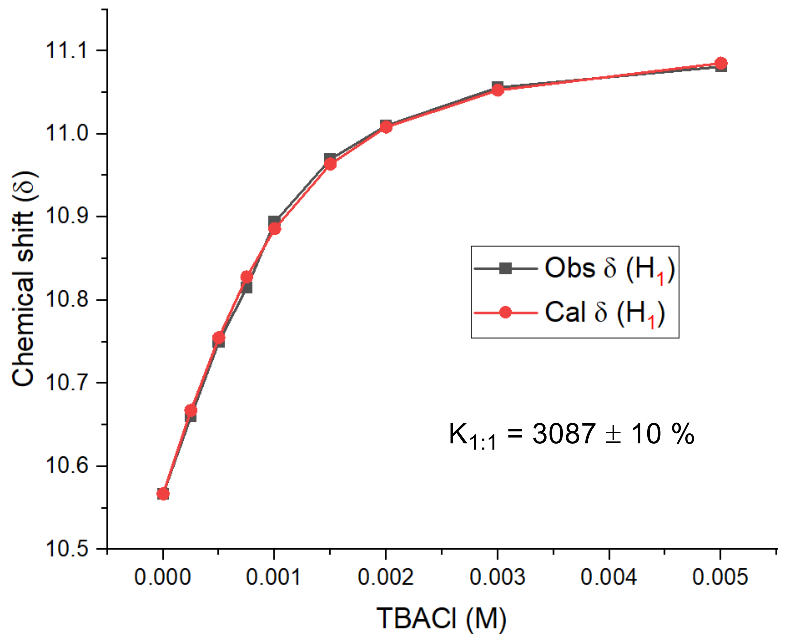

Figure S24. The plot of chemical shift (δ) of H_1 proton *vs* concentration of TBACl added, fitted to 1:1 binding model using BindFit v0.5.

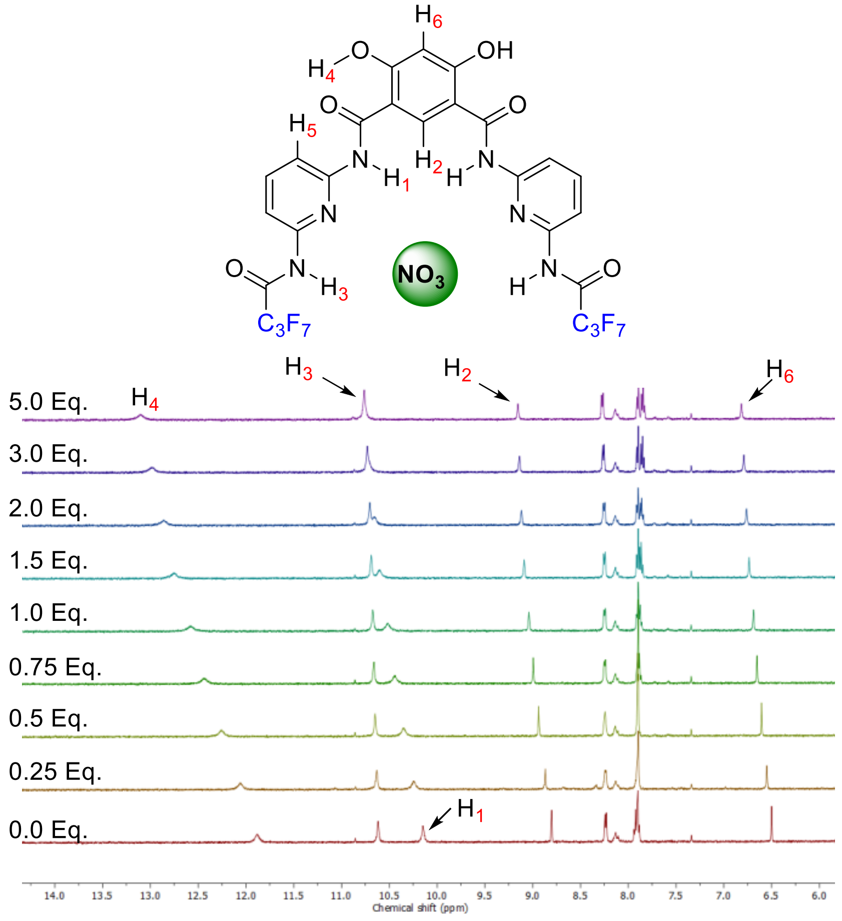

Figure 25. ^{1}H NMR titration spectra for **1a** (1.0 mM in THF-d_8) with stepwise addition of $TBANO_3$ in THF-d_8. The equivalents of added TBACl are reported on the stacked spectra.

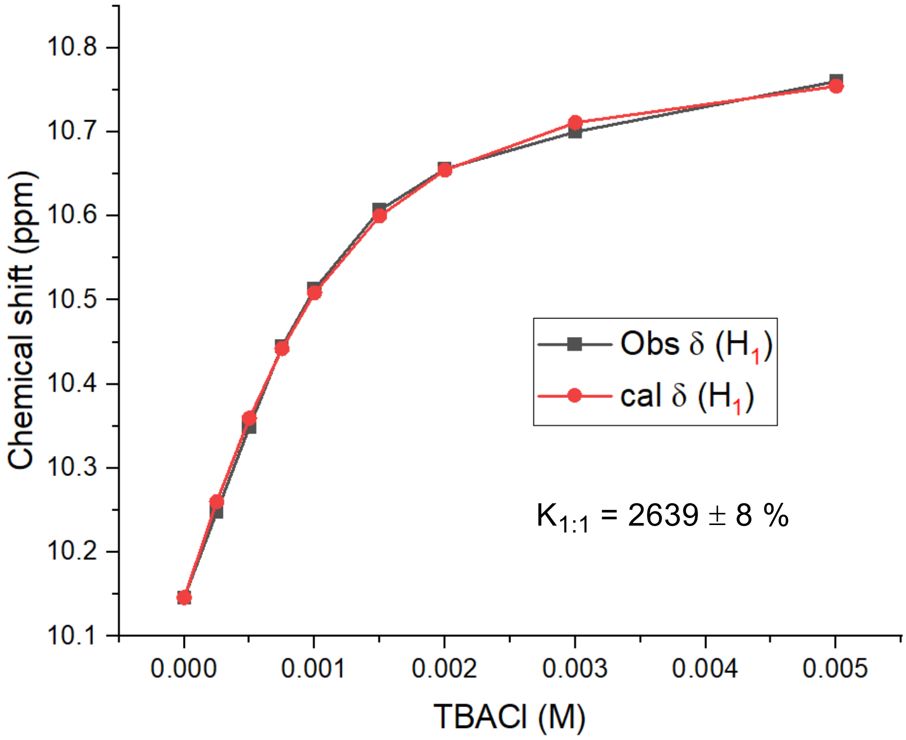

Figure S26. The plot of chemical shift (δ) of H_1 proton *vs* concentration of $TBANO_3$ added, fitted to 1:1 binding model using BindFit v0.5.

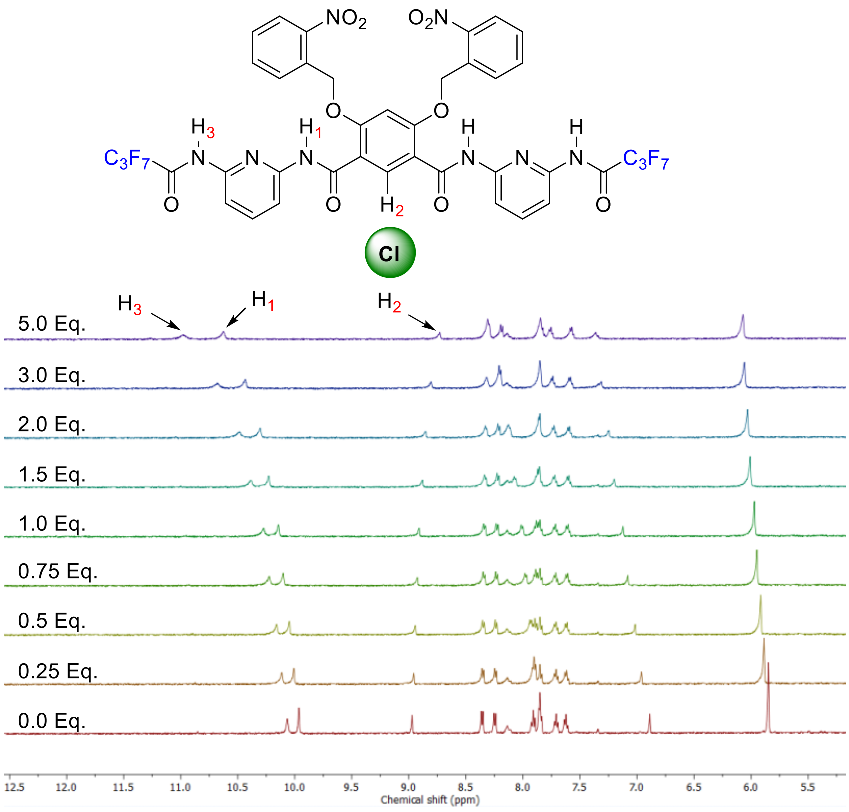

Figure S27. ^{1}H NMR titration spectra for **1a** (2.5 mM in THF-d_8) with stepwise addition of TBACl in THF-d_8. The equivalents of added TBACl are reported on the stacked spectra.

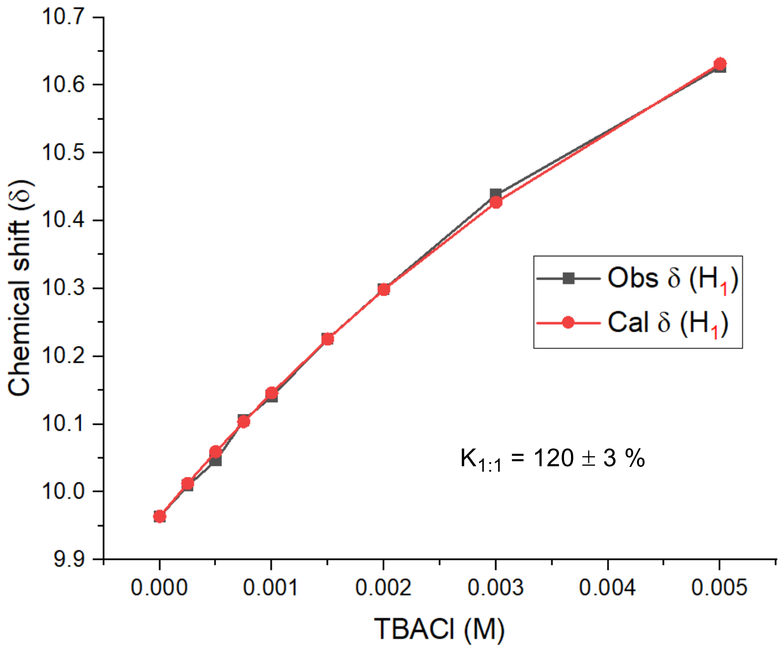

Figure S28. The plot of chemical shift (δ) of H_1 proton *vs* concentration of TBACl added, fitted to 1:1 binding model using BindFit v0.5.

V. Ion transport studies

Vesicle preparation

A thin film of lipid (1-palmitoyl-2-oleoyl-*sn*-3-phosphatidylcholine (POPC) or dipalmitoyl phosphatidylcholine DPPC) was formed by evaporating a chloroform solution under a stream of nitrogen gas, and then under high vacuum for 6 hours. The lipid film was hydrated by vortexing with the prepared buffer (100 mM NaCl, 10 mM HEPES, 1 mM 8-hydroxypyrene-1,3,6-trisulfonic acid trisodium salt (HPTS), pH 7.0). The lipid suspension was then subjected to 5 freeze-thaw cycles using liquid nitrogen and a water bath (40°C), followed by extrusion 19 times through a polycarbonate membrane (pore size 200 nm) at rt. Extrusion was performed at 50°C in the case of DPPC lipids. Extra-vesicular components were removed by size exclusion chromatography on a Sephadex G-25 column with 100 mM NaCl, 10 mM HEPES, pH 7.0. Final conditions: LUVs (2.5 mM lipid); inside 100 mM NaCl, 10 mM HEPES, 1 mM HPTS, pH 7.0; outside: 100 mM NaCl, 10 mM HEPES, pH 7.0. Vesicles for the sodium gluconate assay were prepared by the same procedure, substituting NaCl for NaGluconate in the buffer solution.

Transport assays with HPTS

In a typical experiment, the LUVs containing HPTS (40 µL, final lipid concentration 31.3 µM) were added to buffer (2910 µL of 100 mM NaCl, 10 mM HEPES, pH 7.0) at 25°C under gentle stirring. A pulse of NaOH (30 µL, 0.5 M) was added at 20 s to initiate the experiment. At 100 s the test transporter was added in DMF (5 µL), followed by detergent (40 µL of Triton X-100 in 7:1 (v/v) H_2O-DMSO) at 300 secs to calibrate the assay. The fluorescence emission was monitored at λ_{em} = 510 nm (λ_{ex} = 460/405 nm). The fractional fluorescence intensity (I_{rel}) was calculated from equation (S1), where R_t is the fluorescence ratio at time t, (ratio of intensities

460 nm / 405 nm excitation) R_0 is the fluorescence ratio at time 77 s, and R_d is the fluorescence ratio after the addition of detergent.

$$I_{rel} = \frac{R_t - R_0}{R_d - R_0} \qquad \textbf{(S1)}$$

The fractional fluorescence intensity (I_{rel}) at 290 s just prior to lysis, defined as the fractional activity y, was plotted as a function of the ionophore concentration (x / µM). Hill coefficients (n) and EC_{50} values were calculated by fitting to the Hill equation (S2) equation,

$$y = y_0 + (y_{max} - y_0).\frac{x^n}{EC50^n + x^n} \qquad \textbf{(S2)}$$

where y_0 is the fractional activity in the absence of transporter, $y_{\max}$ is the fractional activity in with excess transporter, x is the transporter concentration in the cuvette.

For all compounds each individual concentration was repeated at-least three times and averaged; error bars represent standard deviations.

Experiments with DPPC lipids were conducted in the same way. For elevated temperature studies, the sample was equilibrated at 45°C (using the Peltier temperature controller) for 5 minutes prior to initiating the experiment.

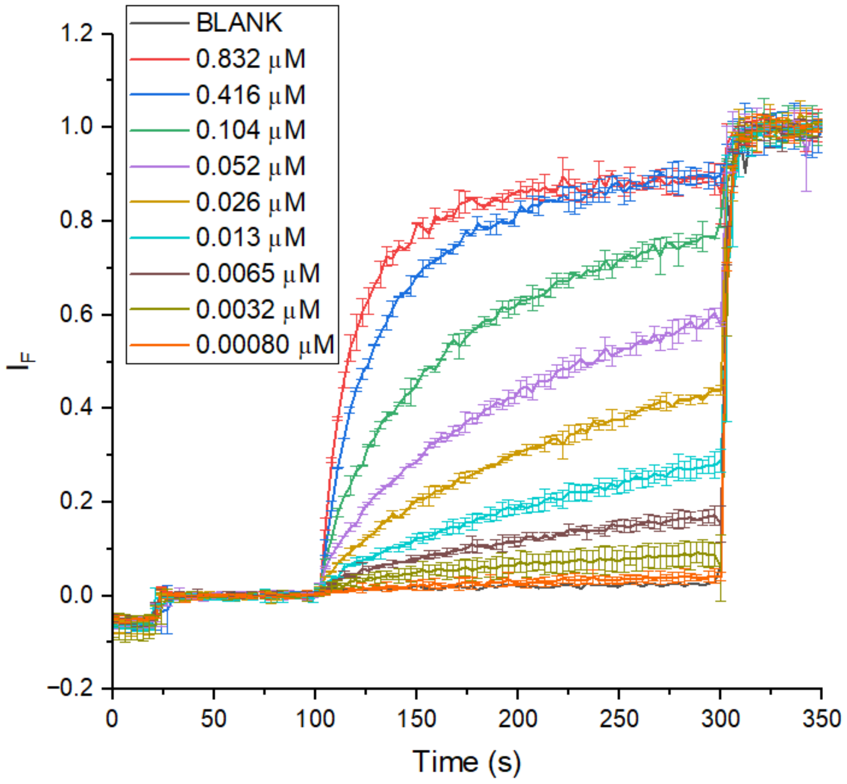

Figure S29. Ion transport HPTS assay data for **1** in POPC LUVs.

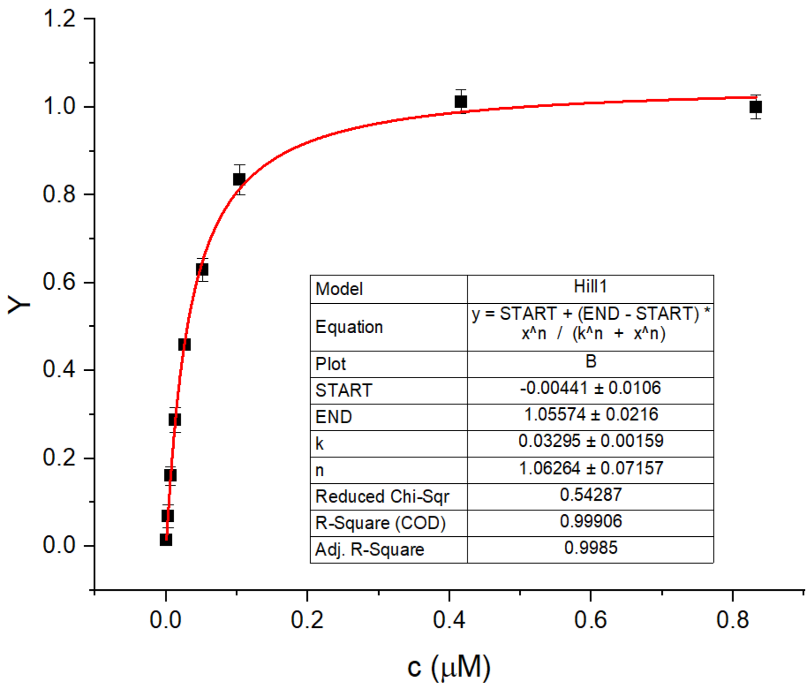

Figure S30. Hill plot for **2**.

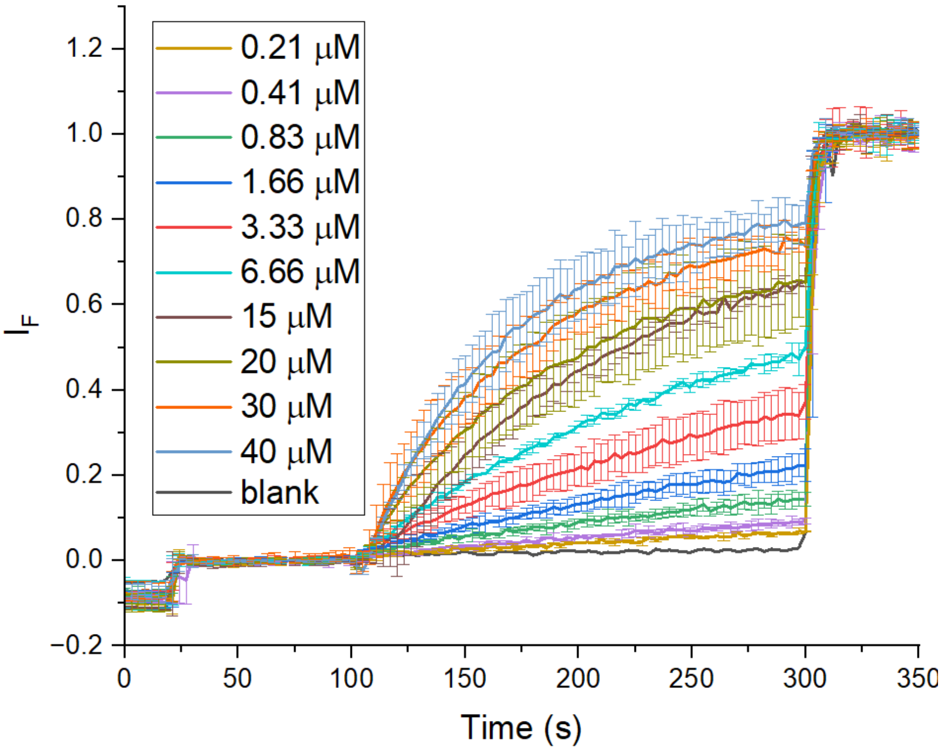

Figure S31. Ion transport HPTS assay data for **2** in POPC LUVs.

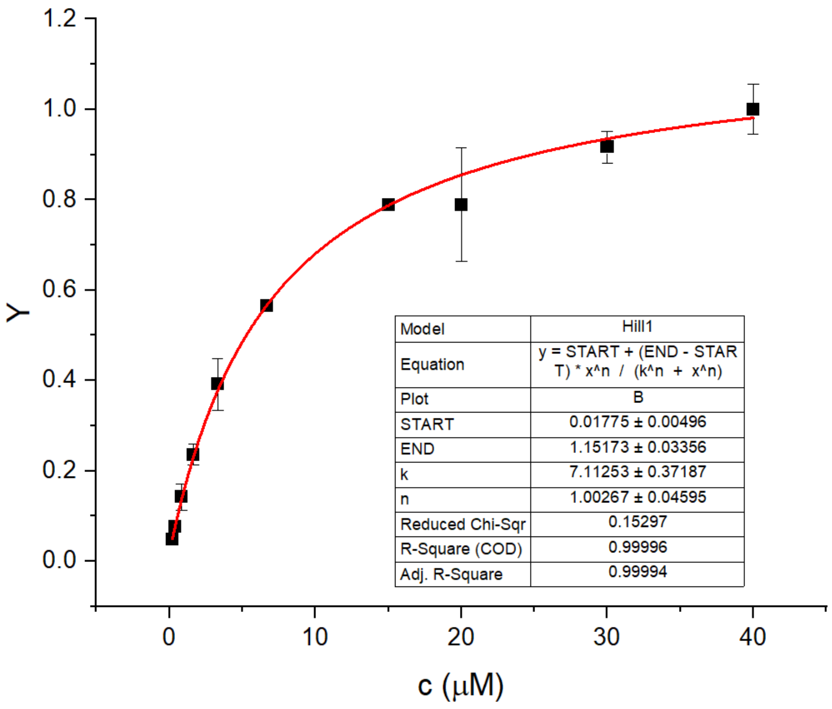

Figure S32. Hill plot for **3**.

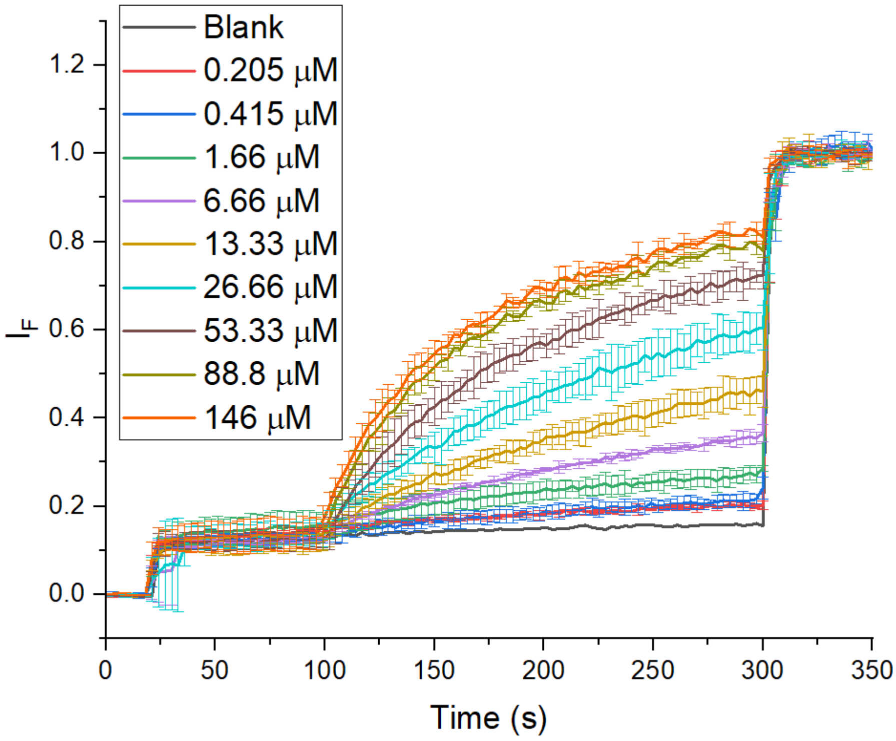

Figure S33. Ion transport HPTS assay data for **3** in POPC LUVs.

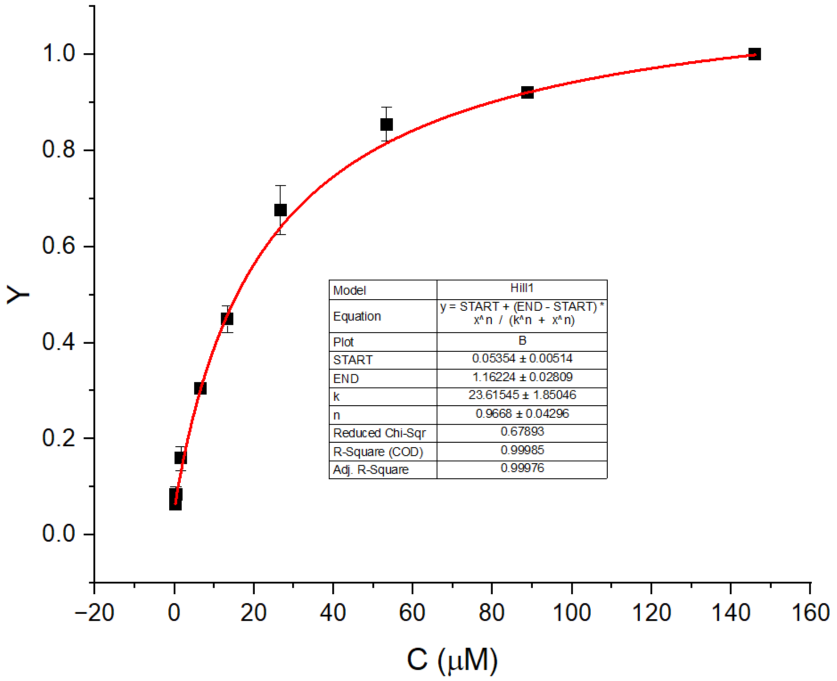

Figure S34. Hill plot for **3**.

Effect of external ion exchange

The effect of external ion exchange was explored by using a previously reported external ion exchange assay.[5-7] These experiments were carried out by adding the POPC vesicle solution (prepared as above) to buffer (100 mM MX, 10 mM HEPES, pH 7.0), where M = Li, Na, K, Rb, Cs (X = Cl), and X = Cl, Br, I, NO_3, and ClO_4 (M = Na).

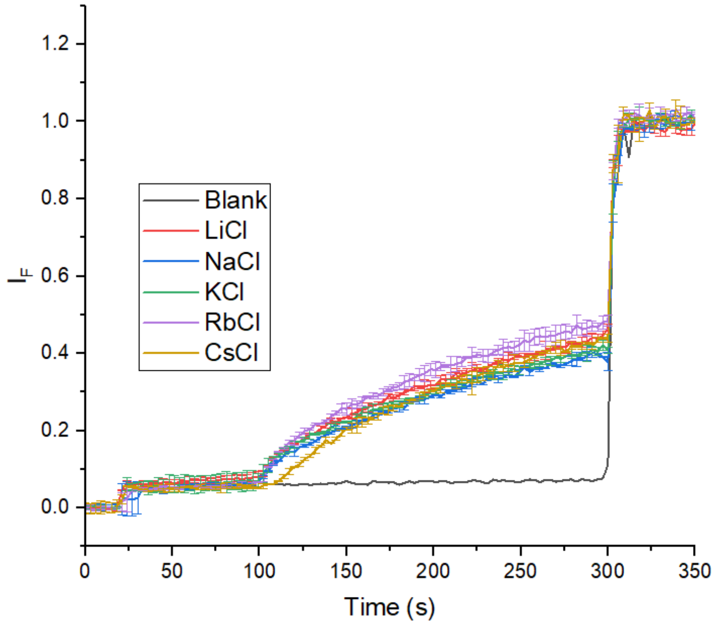

Figure S35. HPTS assay of **1** at 3 mol% carrier concentration and varying external anions (external buffer: 100 mM MCl (M = Li, Na, K, Rb, and Cs, 10 mM HEPES).

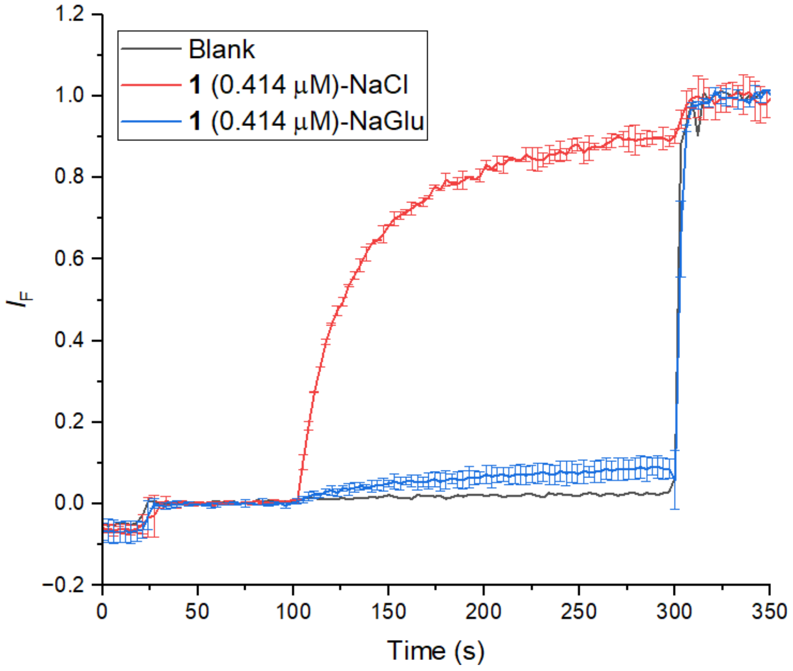

Figure S36. HPTS transport activity of **1** (0.414 μM) in presence of either internal NaCl or Na-Gluconate solutions.

HPTS Assay with DPPC lipids

This HPTS assay was carried out using the same procedure as described above, however, the POPC LUVs were replaced with dipalmitoyl phosphatidylcholine (DPPC) LUVs. These lipids were extruded at 45 °C.

Lucigenin chloride transport assays.

Preparation of POPC-LUVs⊃lucigenin vesicles: In a 10 mL clean and dry round bottom flask, the thin transparent film of 1-palmitoyl-2-oleoyl-*sn*-3-phosphatidylcholine (POPC) was formed by drying 1.0 mL POPC (25 mg/mL in $CHCl_3$) whilst providing continuous rotation and purging nitrogen. The transparent thin film was kept under a high vacuum for 4 hours to remove all traces of $CHCl_3$, before it was hydrated with 1.0 mL aqueous $NaNO_3$ (200 mM, 1.0 mM Lucigenin) buffered to pH 6.5 using 10 mM phosphate buffer, with occasional vortexing at 10 min intervals for 1 h. The resulting suspension was subjected to freeze and thaw cycles (≥ 10 liquid nitrogen, 55 °C water bath) and 21 times extrusion through 200 nm pore size polycarbonate membrane. Size exclusion chromatography (using Sephadex G-50) was performed to remove extravesicular dye using 200 mM $NaNO_3$ solution as eluent. The collected vesicles suspension was diluted to 4 mL. Final conditions: ~ 5 mM POPC; inside: 200 mM $NaNO_3$, 1 mM lucigenin, 10 mM phosphate buffer at pH 6.5; outside: 200 mM $NaNO_3$, 10 mM phosphate buffer at pH 6.5.

Ion transport activity across Lucigenin-containing vesicles:

To perform the experiment, in clean and dry fluorescence cuvette, 200 mM $NaNO_3$ solution buffered at 6.5 (2910 μL), POPC-LUVs⊃lucigenin (20 μL, 54.8 μM) and ion transporter **1**

(varying conc, 20 µL from DMF solution) were added. This suspension was placed in a slowly stirring condition in a fluorescence instrument equipped with a magnetic stirrer (at $t = 0$ s). The fluorescence intensity of lucigenin was monitored at $\lambda_{em} = 535$ nm ($\lambda_{ex} = 455$ nm) over time. The chloride gradient was created by the addition of 2.0 M NaCl (50 µL) at $t = 50$ s between intra- and extravesicular solutions. Finally, vesicles were lysed by adding 10% Triton X-100 (40 µL) at $t = 150$ s for the complete destruction of the chloride gradient.

The time-dependent data were normalized to percent change in fluorescence intensity using Equation S4:

$$I_F = [(I_t - I_0) / (I_\infty - I_0)] \times (-1) \qquad \text{Equation S4}$$

where, I_0 is the initial intensity, I_t is the intensity at time t, and I_∞ is the final intensity after addition of Triton X-100.

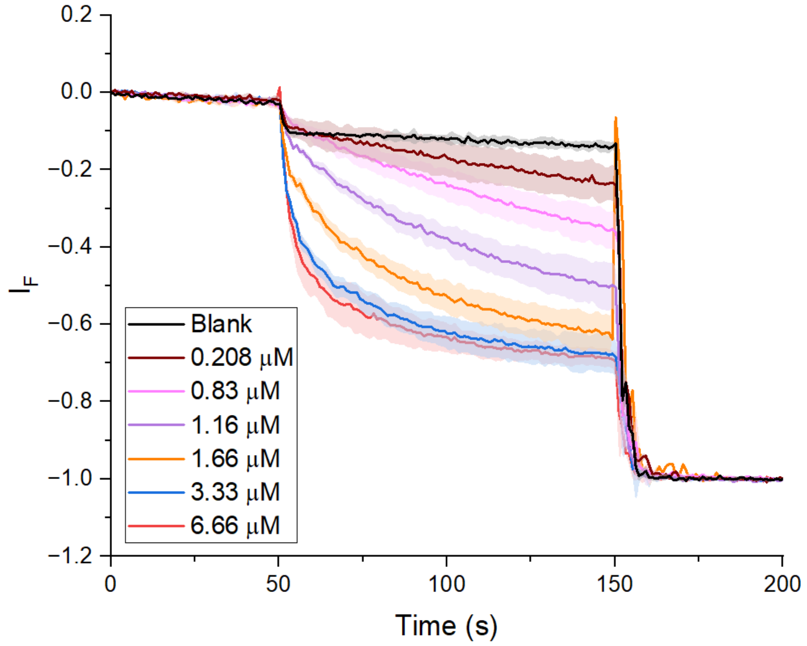

Figure S37. Ion transport lucigenin assay data for **1** in POPC LUVs.

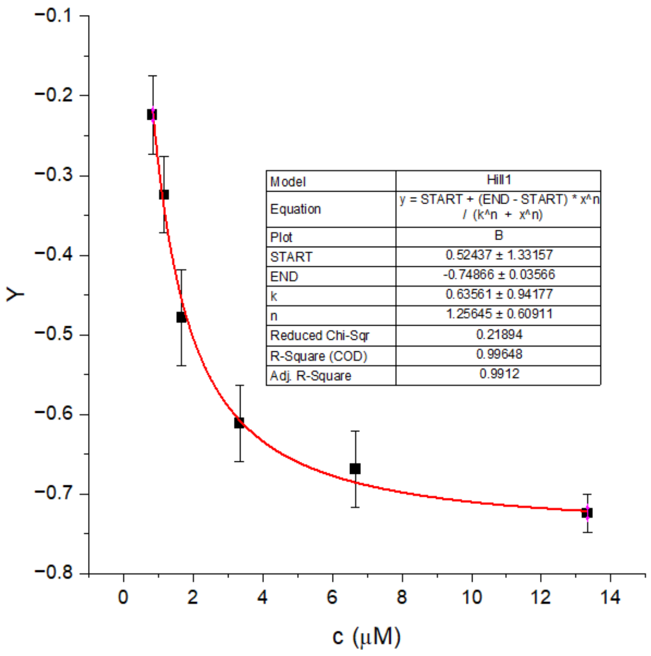

Figure S38. Hill plot for **1**.

Lucigenin assay in the presence of external SO_4^{2-} and NO_3^- anions

Preparation of POPC-LUVs⊃lucigenin vesicles: In a 10 mL clean and dry round bottom flask, the thin transparent film of 1-palmitoyl-2-oleoyl-sn-glycero-3-phosphocholine (POPC was formed by drying 1.0 mL POPC (25 mg/mL in $CHCl_3$) while providing continuous rotation and purging nitrogen. The transparent thin film was kept under a high vacuum for 4 hours to remove all traces of $CHCl_3$. Then the transparent thin film was hydrated with 1.0 mL aqueous NaCl (200 mM, 1.0 mM Lucigenin) buffered at pH of 6.5 with 10 mM phosphate, with occasional vortexing at 10 min intervals for 1 h. The resulting suspension was subjected to freeze and thaw cycles (≥ 10 liquid nitrogen, 55 ° C water bath) and 21 times extrusion through 200 nm pore size polycarbonate membrane. The size exclusion chromatography (using Sephadex G-50) was performed to remove extravesicular dye using 200 mM NaCl solution as eluent. The collected vesicles suspension was diluted to 4 mL. Final conditions: ~ 5 mM POPC; inside: 200 mM NaCl, 1 mM lucigenin, 10 mM phosphate buffer at pH 6.5; outside: either 200 mM $NaNO_3$ or 200 mM Na_2SO_4 and 10 mM phosphate buffer at pH 6.5.

Ion transport assay

In a clean and dry fluorescence cuvette, either 200 mM $NaNO_3$ or Na_2SO_4 solution buffered with phosphate, pH = 6.5 (2890 μL) and POPC-LUVs⊃lucigenin (40 μL, 109.6 μM) was added. This suspension was placed in a slowly stirring condition in a fluorescence instrument equipped with a magnetic stirrer (at t = 0 s). The fluorescence intensity of lucigenin was monitored at λ_{em} = 535 nm (λ_{ex} = 455 nm) as a course of time. The transporter molecule **1** (3.0 mol%) was added at t = 50 s as a solution in DMF (5 μL, 2 mM). Finally, vesicles were lysed by adding 10% Triton X-100 (40 μL) at t = 250 s for the complete destruction of chloride gradient. NO_3^- transport occurred with the concomitant efflux of Cl^- ions, whilst, in contrast,

transport was suppressed with the more hydrophilic SO_4^{2-} anion which is not readily transported, suggesting the operation of a Cl^-/NO_3^-antiport mechanism.

The time-dependent data were normalized to percent change in fluorescence intensity using Equation S3.

$$I_F = [(I_t - I_0) / (I_\infty - I_0)] \times (-1) \qquad \text{Equation S3}$$

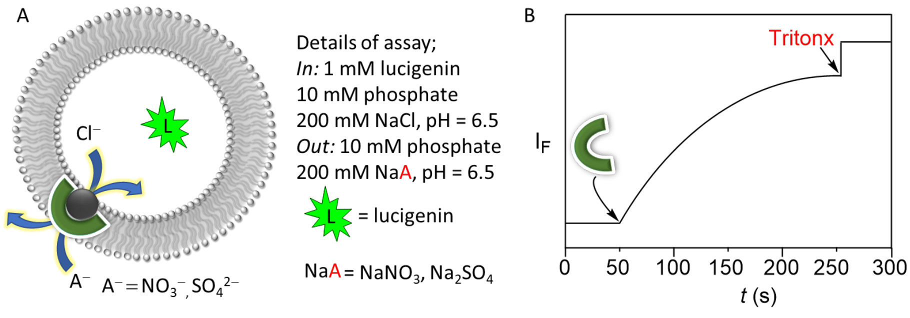

Figure S39. Representation of fluorescence-based antiport assay using POPC-LUVs⊃lucigenin (A) Representation of ion transport kinetics showing normalization window

VI. Solution phase photocleavage and ligand binding studies

Assessment of photolysis of protransporter 1a to give 1 upon irradiation at 405 nm: In a clean and dry NMR tube, the solution of **1a** was taken in DMSO-d_6 (1 mM in 0.5 mL). The ^{1}H NMR spectrum of the sample was recorded (t = 0 min) and then the NMR tube containing the compound **1a** was photoirradiated using 405 nm LED (1W) for different time intervals. The ^{1}H NMR spectrum of the irradiated samples were recorded at the end of each irradiation. All ^{1}H NMR spectra were processed using MestReNova 6.0 by considering the residual solvent peak as an internal reference. Finally, the NMR spectra of **1a** and the photoirradiated samples were stacked and compared with as synthesized **1** (recorded in DMSO-d_6). Upon photoirradiation, the appearance and disappearance of the different proton peak signals of the protransporter **1a** indicated the release of as-synthesized active transporter **1**.

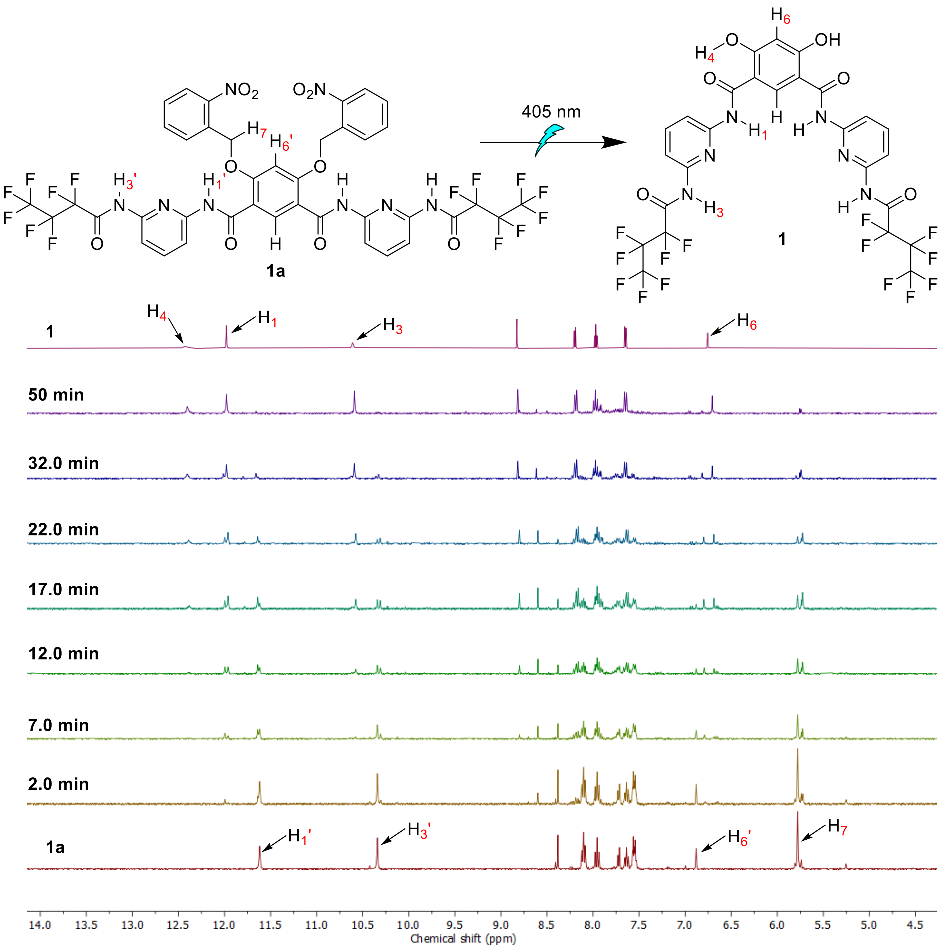

Figure S40. Photo release of active transporter **1** from pro-transporter **1a** upon photoirradiation using 405 nm LEDs (1 W) recorded in DMSO-d_6.

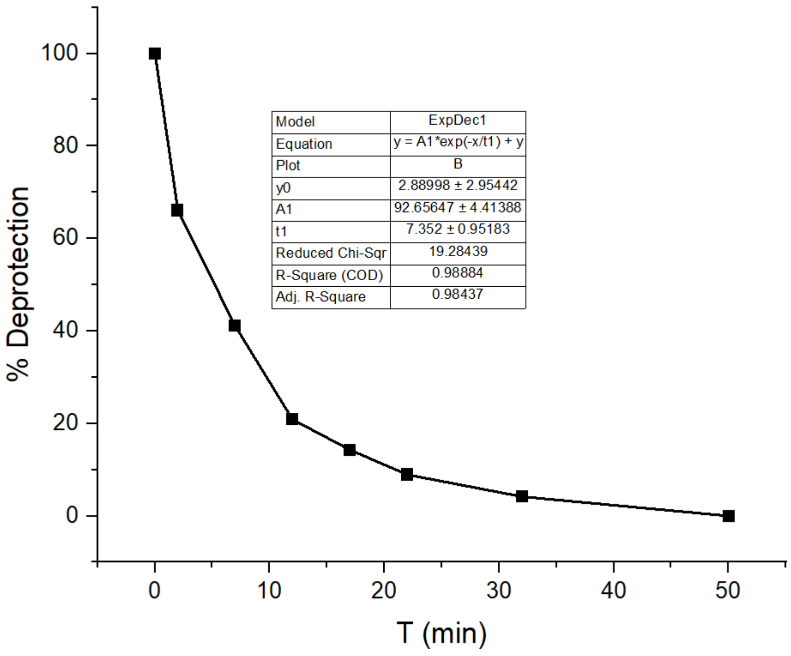

Figure S41. % deprotection of **1a** after irradiation with 405 nm light using 1 W LED.

UV-visible titration studies of 1 with barbiturate B1 and TBACl

UV-visible absorption titration experiments were performed at 298 K using a Jasco V-770 UV-visible/NIR spectrophotometer equipped with a Peltier temperature controller. Initially stock solutions of transporter **1** (1 mM), barbiturate ligand **B1** (200 mM) and TBACl (1000 mM) were prepared in DMF. Subsequently, aliquots of either **B1** using 200 mM stock solution were added to transporter **1** (6.6 µM, using 1 mM stock solution) or TBACl using 1000 mM stock solutions were added to transporter **1** (3.3 µM, using 1 mM stock solution) in 3.0 mL of a quartz glass cuvette and UV-vis spectra was recorded after each addition. The absorbance intensities at 359 nm for barbiturate addition and 396 for TBACl addition were plotted, and the resulting isotherms fitted to a 1:1 stoichiometric host-guest binding model using Bindfit.

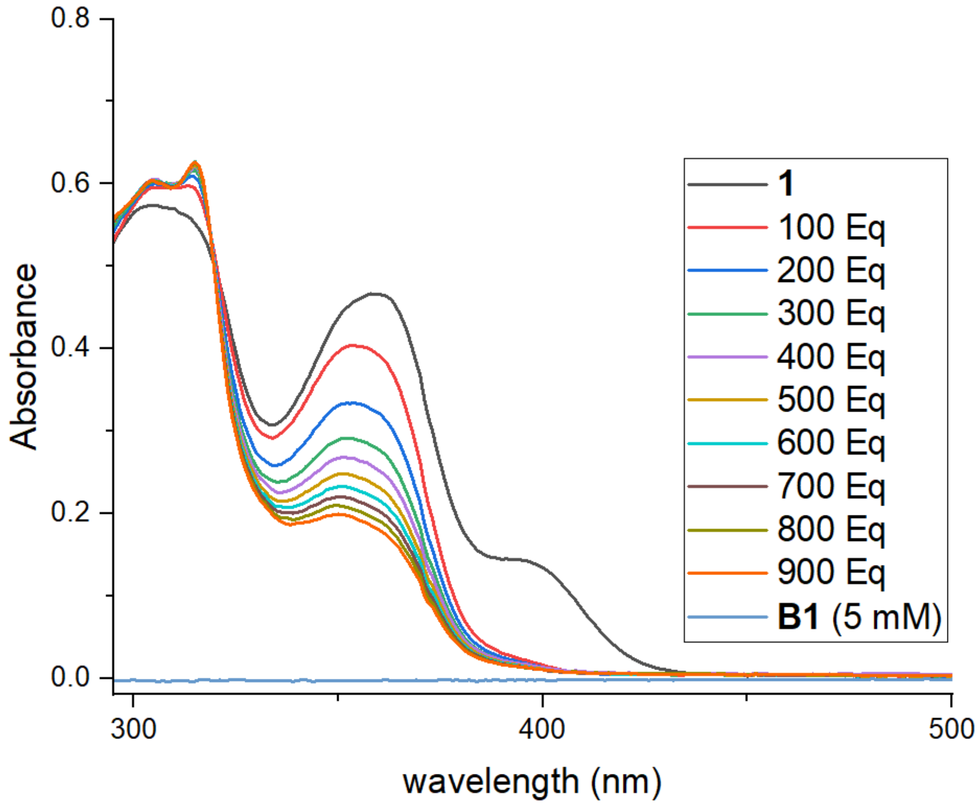

Figure S42. UV-Visible spectra of transporter **1** (6.6 µM, DMF) upon successive addition of barbiturate ligand **B1**. Black arrows indicate direction of change.

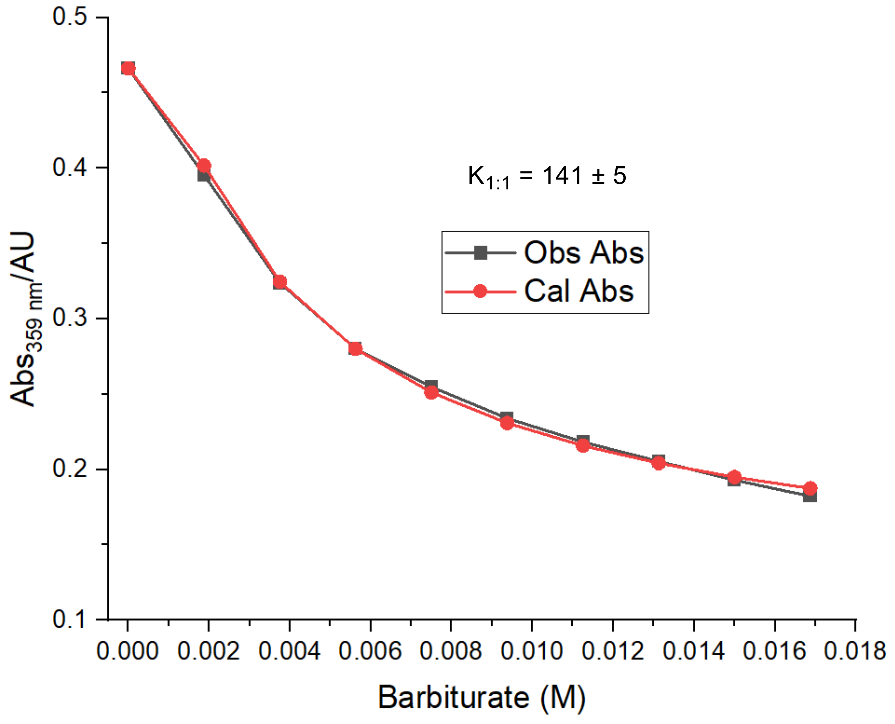

Figure S43. The plot of absorbance (359nm) for **1** *vs* concentration of barbiturate (**B1**) added, fitted to 1:1 binding model of BindFit v0.5.

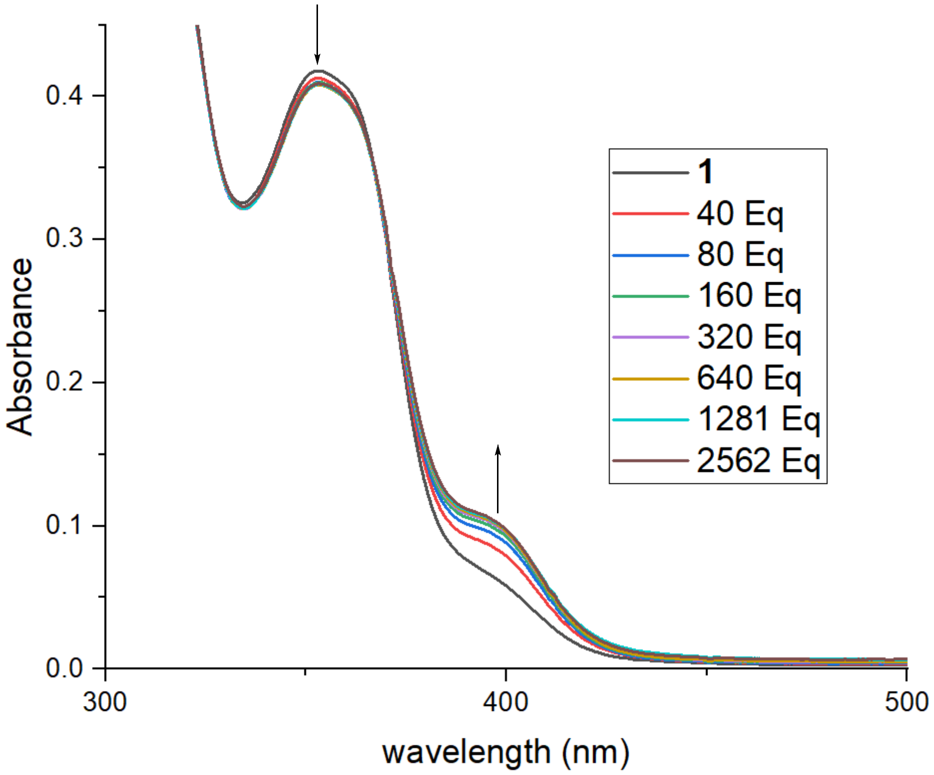

Figure S44. UV-Visible spectra of transporter **1** (3.3 μM, DMF) upon successive addition of TBACl. Black arrows indicate direction of change.

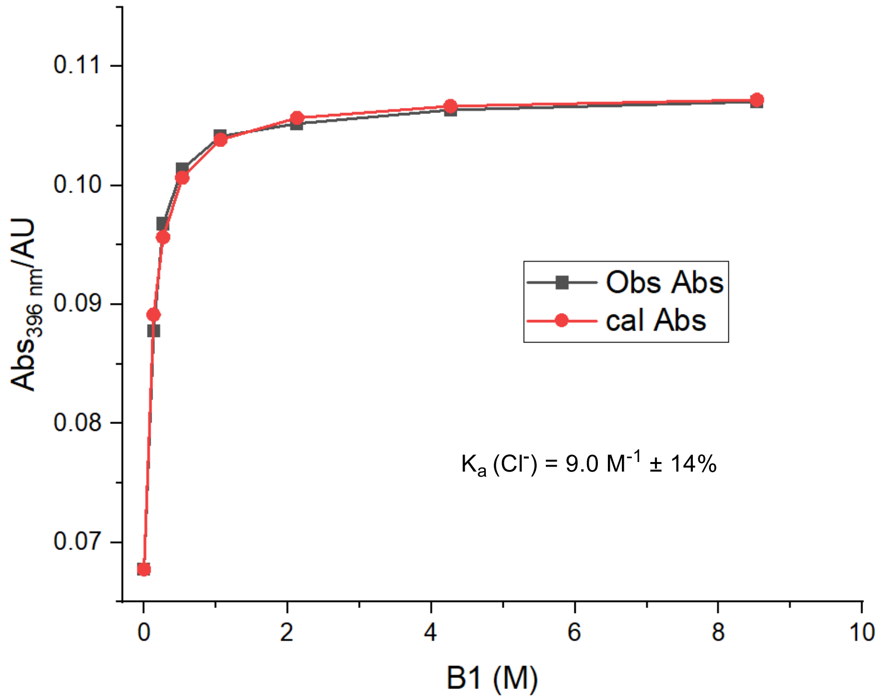

Figure S45. The plot of absorbance (396nm) for **1** *vs* concentration of TBACl added, fitted to 1:1 binding model of BindFit v0.5.

VII. Stimulus-responsive ion transport studies

Preparation of POPC-LUVs⊃lucigenin vesicles: These vesicles were prepared using the procedure as described above.

Activation with light:

In a clean and dry fluorescence cuvette, 200 mM of $NaNO_3$ (2890 µL), POPC-LUVs⊃lucigenin (40 µL, final concentration 109.6 µM), and **1a** (5 µL from DMF solution, final concentration of 3.3 µM) were added. The fluorescence intensity of lucigenin was monitored at λ_{em} = 535 nm (λ_{ex} = 455 nm) over time. A chloride gradiant was generated across the membrane by adding NaCl at t = 50 s in the external buffer. At the end of the experiment the LUVs were lysed by adding 10% Triton X-100 (40 µL) at t = 250 s for the complete destruction of chloride gradient. Negligible transport activity was observed for **1a**. The buffer containing **1a** was then initially photoirradiated at 405 nm light using an LED (1W) and subsequently POPC-LUVs⊃lucigenin (40 µL, final concentration 109.6 µM) were added and ion transport was monitored. The same procedure was employed for photoirradiation at different time intervals and at each time vesicles were added after the photoirradiation process.

The time-dependent data were normalized to percent change in fluorescence intensity using Equation S1.

Deactivation with barbiturate ligand B1:

In a clean and dry fluorescence cuvette, 200 mM of $NaNO_3$ (2890 µL), and **1a** (3.3 µM) were added. This solution was then photoirradiated at 405 nm of light using LEDs for 5 minutes to generate **1**, and subsequently barbiturate **B1** (using 50 mM stock solution in DMF) and POPC-LUVs⊃lucigenin (40 µL, 109.6 µM) were added and ion transport was monitored.

The time-dependent data were normalized to percent change in fluorescence intensity using Equation S1. **B1** has a clogP ~0.7 (P ~5), so assuming that log P is a reasonable measure of membrane partitioning, it is likely that the hydrophilicty of **B1** resulst in relatively poor membarne uptake, hence a large excess is required to fully saturate **1** within the membrane and inhibit anion transport.

VIII. References

1. P. Tecilla, V. Jubian and A. D. Hamilton, Synthetic hydrogen bonding receptors as models of transacylase enzymes, *Tetrahedron*, 1995, **51**, 435-448.
2. E. Kolomiets, V. Berl and J. M. Lehn, Chirality induction and protonation-induced molecular motions in helical molecular strands, *Chemistry*, 2007, **13**, 5466-5479.
3. http://app.supramolecular.org/bindfit/
